# Asymmetrical Interactions between Ni Single Atomic Sites and Ni Clusters in a 3D Porous Organic Framework for Enhanced CO_2_ Photoreduction

**DOI:** 10.1002/advs.202401508

**Published:** 2024-03-15

**Authors:** Fang‐Qin Yan, Xiao‐Yu Dong, Yi‐Man Wang, Qian‐You Wang, Shan Wang, Shuang‐Quan Zang

**Affiliations:** ^1^ Henan Key Laboratory of Crystalline Molecular Functional Materials and College of Chemistry Zhengzhou University Zhengzhou 450001 China

**Keywords:** CO_2_ photoreduction, metal nanoclusters, porous organic frameworks, single‐atoms

## Abstract

3D porous organic frameworks, which possess the advantages of high surface area and abundant exposed active sites, are considered ideal platforms to accommodate single atoms (SAs) and metal nanoclusters (NCs) in high‐performance catalysts; however, very little research has been conducted in this field. In the present work, a 3D porous organic framework containing Ni_1_ SAs and Ni_n_ NCs is prepared through the metal‐assisted one‐pot polycondensation of tetraaldehyde and hexaaminotriptycene. The single metal sites and metal clusters confined in the 3D space created a favorable micro‐environment that facilitated the activation of chemically inert CO_2_ molecules, thus promoting the overall photoconversion efficiency and selectivity of CO_2_ reduction. The 3D‐NiSAs/NiNCs‐POPs, as a CO_2_ photoreduction catalyst, demonstrated an exceptional CO production rate of 6.24 mmol g^−1^ h^−1^, high selectivity of 98%, and excellent stability. The theoretical calculations uncovered that asymmetrical interaction between Ni_1_ SAs and Ni_n_ NCs not only favored the bending of CO_2_ molecules and reducing the CO_2_ reduction energy, but also regulated the electronic structure of the catalyst leading to the optimal binding strength of intermediates.

## Graphical abstract

Single Ni_1_ sites and Ni_n_ clusters simultaneously incorporating into one 3D porous organic framework is reported. Experimental results and theoretical calculations demonstrated that the asymmetrical interactions between of SAs and NCs confined in 3D space led to favorable micro‐environments for the activation of chemically inert CO_2_, leading to enhanced CO_2_ photocatalytic reduction efficiency.

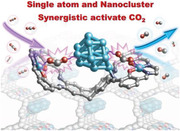

## 1 Introduction

The ambition to achieve the carbon neutrality goal by 2050 has driven the development of advanced technologies to convert CO_2_ into value‐added chemicals.^[^ ^1^ ^]^ Photocatalytic CO_2_ reduction reaction (CO_2_RR) have been recognized as eco‐friendly and sustainable ways because they are clean, noninvasive, and remotely controllable.^[^ ^2^ ^]^ Although significant achievements have been made in optimizing the light harvesting and charge separation of photocatalysts,^[^ ^3^ ^]^ the adsorption/activation of CO_2_ on catalysts is generally ignored, greatly limiting the overall photoconversion efficiency and selectivity of CO_2_ reduction. CO_2_ molecules are chemically inert, and their C═O bonds possess a high dissociation energy of ≈750 kJ mol^−1^; thus, highly efficient catalysts are required to activate and convert CO_2._^[^ ^4^ ^]^ In CO_2_ molecules, O atoms with a lone pair of electrons can donate Lewis acid centers, whereas C atoms can accept electrons from Lewis base centers.^[^ ^5^ ^]^ Therefore, the formation of a neighboring synergistic micro‐environment in catalysts to bend the linear molecular structure of CO_2_ and break C═O bonds can be an efficient approach to activate and reduce CO_2_.

Among current CO_2_RR catalysts, single atoms (SAs)‐based catalysts with maximum atomic utilization and a tunable electronic structure have received considerable attention; however, the high surface energy of SAs leads to a low density of active sites to activate CO_2._^[^ ^6^ ^]^ Metal nanoclusters (NCs) with a high surface‐to‐volume ratio and abundant exposed active sites have recently been identified as important nano‐catalysts for CO_2_RR;^[^ ^7^ ^]^ however, the stronger adsorption of substrates often results in less activity.^[^ ^8^ ^]^ Therefore, the integration of SAs with NCs to modulate the adsorption and activation of CO_2_ molecules can greatly promote the CO_2_ photoreduction performance of catalysts. Although reported nanocluster‐single atom (NC‐SA) catalysts exhibit excellent photocatalytic CO_2_RR performance,^[^ ^9^ ^]^ research on the critical roles of SAs and NCs during CO_2_ activation processes is absent. Moreover, the synthesis of NC‐SA catalysts is often assisted by pyrolysis, resulting in vague SA and NC micro‐environments and an unclear structure‐reactivity relationship.^[^ ^10^ ^]^

Porous organic polymers (POPs) are generally linked by covalent bonds and have predesigned structures with well‐developed pores; thus, they can be ideal platforms to accommodate SAs and NCs for photocatalytic CO_2_RR.^[^ ^11^ ^]^ Particularly, porous architectures can accelerate the diffusion of CO_2_ molecules to internal active sites and also the migration of intermediates and products, thereby resulting in fast reaction kinetics.^[^ ^12^ ^]^ In our previous work, a 2D POP‐containing salphen units were used to form SA active sites with an N_2_O_2_ coordination mode for efficient CO_2_ photoreduction.^[^ ^13^ ^]^ Deng et al. demonstrated the high CO_2_ to CO photocatalytic activity of single metal‐salphen sites in a covalent organic framework.^[^ ^14^ ^]^ Different from 2D counterparts, 3D porous materials possessing higher surface area and larger voids,^[^ ^15^ ^]^ play a crucial role in accommodating SAs and NCs in catalysts. Ni SACs have been extensively studied for catalysis due to its high abundance, diverse redox properties, and moderate reactant adsorption capability.^[^ ^16^ ^]^ Several pioneering works have also demonstrated its exceptional activity and selectivity for conversion CO_2_ to CO. For example, Li et al. develops a Ni SACs by ZIF assisted strategy, which are capable of selectively reducing CO_2_ with excellent faraday efficiency.^[^ ^17^ ^]^ Liu et al. constructed a low‐valent Ni(I) SACs with high intrinsic CO_2_ reduction activity.^[^ ^18^ ^]^ Therefore, 3D POPs with Ni_1_−N_2_O_2_ sites and Ni_n_ NCs can be perfect candidates for promoting the adsorption and activation of CO_2_.

In the current work, 2,3,6,7,14,15‐hexaaminotriptycene hexahydro chloride (HATT) was employed as a rigid 3D building block, which reacted with 3,3′,5,5′‐tetraformyl‐4,4′‐biphenyldiol (TFBD) and metal sources to obtain a 3D POP (denoted as 3D‐NiSAs/NiNCs‐POPs). The as‐prepared framework contained salphen units to anchor Ni SA sites with an explicit ‐N_2_O_2_ coordination mode and also possessed electron‐donor atoms and a suitable void space to confine NCs. The 3D‐NiSAs/NiNCs‐POPs exhibited a fascinating CO_2_ to CO photocatalytic activity (31 200 µmol g^−1^) and high selectivity (98%) under visible light irradiation for 5 hours, and this performance is far superior to SAs counterpart 3D‐NiSAs‐POPs and those of reported Ni‐based porous photocatalysts. The synergistic contribution of Ni_1_ SAs and Ni_n_ NCs in the 3D POP was disclosed by density functional theory (DFT) calculations, and efficient activation of CO_2_ molecules was realized by asymmetrically binding two O atoms by Ni_1_ SAs and Ni_n_ NCs. Moreover, the distinct electronic configurations of NCs and SAs led to an optimal binding strength of intermediates and accelerated the kinetic process.

## 2 Results and Discussion

The one‐pot solvothermal reaction of TFBD, HATT, and Ni(OAc)_2_·H_2_O with N, N‐dimethylformamide (DMF) at 100 °C yielded a dark red powder of 3D‐NiSAs/NiNCs‐POPs.

**Figure 1:**
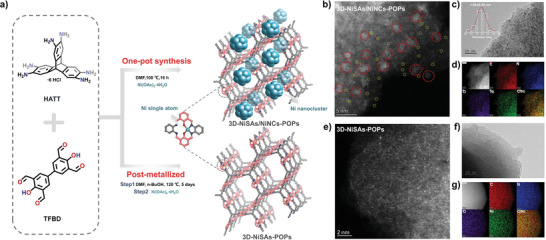
a) Schematic illustration of the synthesis routes of 3D‐NiSAs/NiNCs‐POPs and 3D‐NiSAs‐POPs. The depicted structures are the ideal models. b, e) Atomic‐resolution HAADF‐STEM images of 3D‐NiSAs/NiNCs‐POPs and 3D‐NiSAs‐POPs, respectively. c, f) TEM images of 3D‐NiSAs/NiNCs‐POPs and 3D‐NiSAs‐POPs, respectively (inset in Figure 1c shows the average size distribution of Ni NCs). d, g) Corresponding EDS mapping images of 3D‐NiSAs/NiNCs‐POPs and 3D‐NiSAs‐POPs, respectively.

The schematic illustration of the synthesis route of 3D‐NiSAs/NiNCs‐POPs is presented in **Figures** **1**a and S1 (Supporting Information). The powder X‐ray diffraction (PXRD) pattern only had a board peak at ≈24°, indicating that the obtained material was amorphous and possessed no large metal particles and nickel salt residues (Figure S2, Supporting Information). The atomic‐resolution high‐angle annular dark‐field scanning transmission electron microscopy (HAADF‐STEM) image in Figure 1b and the transmission electron microscopy (TEM) image in Figure 1c reveal the coexistence of the bright spots of Ni NCs and Ni SAs. On the one hand, the polycondensation of −NH_2_ and −CHO formed salphen pockets, which anchored single Ni sites with a −N_2_O_2_ coordination mode. On the other hand, DMF may reduce the partial metal sources and made them grow into NCs.^[^ ^19^ ^]^ A control experiment was conducted using 1,4‐dioxane as the solvent. As shown in Figure S3 (Supporting Information), no significant NCs were observed in the TEM image, further supporting that DMF function as the reducing agent facilitate the generation of the Ni NCs. A charge density accumulation around Ni NCs with a depletion of the charge density surrounding N and O atoms was observed, indicating the migration of electrons from N and O to Ni atoms (Figure S4, Supporting Information). This result indicated that the unique 3D framework could stabilize the Ni NCs. Figure 1c showed that Ni NCs with an average size of 1.92 ± 0.45 nm were evenly distributed on the POP. Moreover, the energy‐dispersive X‐ray spectroscopy (EDS) results in Figure 1d reveal that C, N, O, and Ni were well distributed over the 3D‐NiSAs/NiNCs‐POPs.

For comparison, a similar solvothermal route with the condensation of TFBD and HATT was developed to prepare 3D‐POPs. Subsequently, the salphen units in this 3D‐POPs were post‐metalized with Ni^2+^ to obtain an SA‐based POP catalyst (denoted as 3D‐NiSAs‐POPs). The atomic‐resolution HAADF‐STEM image in Figure 1e and the TEM image in Figure 1f indicate the uniform distribution of Ni SAs in 3D‐NiSAs‐POPs and there is no visible NCs. The scanning electron microscopy (SEM) images in Figures S6−S8 (Supporting Information) display that all samples contained spherical agglomerates. The thermogravimetric analysis (TGA) results in Figure S9 (Supporting Information) confirm that all materials had good thermal stability with a weight loss of ≈10% at 200 °C. The inductively coupled plasma mass spectrometry (ICP‐MS) results in Table S1 (Supporting Information) state that the Ni contents in 3D‐NiSAs/NiNCs‐POPs and 3D‐NiSAs‐POPs were 10.00 wt% and 3.01 wt%, respectively.

**Figure 2:**
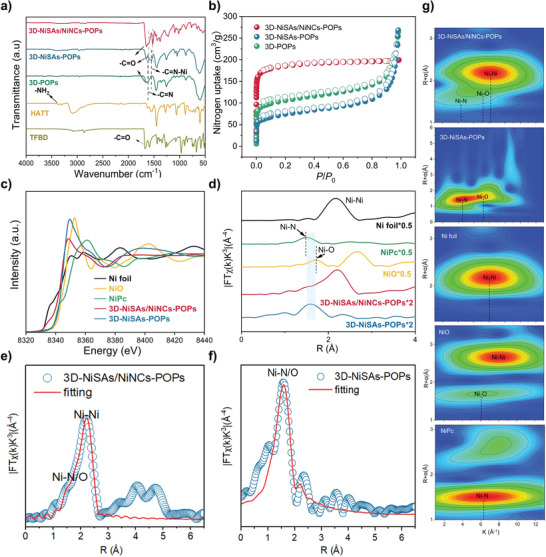
a) FT‐IR spectra of 3D‐NiSAs/NiNCs‐POPs, 3D‐NiSAs‐POPs, 3D‐POPs, HATT and TFBD. b) N_2_ adsorption–desorption isotherm of 3D‐NiSAs/NiNCs‐POPs, 3D‐NiSAs‐POPs and 3D‐POPs. c) XANES and d) EXAFS spectra of 3D‐NiSAs/NiNCs‐POPs, 3D‐NiSAs‐POPs and the reference samples. e, f) EXAFS fitting curves for 3D‐NiSAs/NiNCs‐POPs and 3D‐NiSAs‐POPs in the R‐space. g) WT‐EXAFS contour plots of 3D‐NiSAs/NiNCs‐POPs and 3D‐NiSAs‐POPs, the Ni foil, NiO and NiPc.

Fourier transform infrared (FT‐IR) spectroscopy was carried out to explicate the −N_2_O_2_ Schiff base structural feature of 3D‐NiSAs/NiNCs‐POPs (**Figure** **2**a). The characteristic peaks at 1665 cm^−1^ corresponding to C═O bonds of aldehydes and 3100–3360 cm^−1^ resulting from the N‐H vibration of amine monomers disappeared. A new −C═N stretching vibration characteristic peak appeared at 1611 cm^−1^, indicating the formation of imine bonds.^[^ ^20^ ^]^ In addition, another new peak corresponding to −C═N−Ni was detected at 1530 cm^−1^, implying the coordination of Ni atoms with imine and hydroxyl groups.^[^ ^13^, ^21^ ^]^ The formation of imine linkages in 3D‐POPs and 3D‐NiSAs‐POPs was also demonstrated by FT‐IR. It was found that the −C═N and −C═N−Ni peaks of 3D‐NiSAs/NiNCs‐POPs experienced a subtle redshift due to the introduction of Ni_n_ NCs.^[^ ^22^ ^]^

In order to gain an insight into the coordination environment and chemical state of Ni atoms in the as‐prepared catalysts, X‐ray photoelectron spectroscopy (XPS) was performed (Figure S10,S11, Supporting Information). The O 1s spectra of the metalized catalysts were split into two configurations containing Ni−O and C−O.^[^ ^23^ ^]^ The N 1s profiles of the samples were deconvoluted into C═N−C and C═N−Ni.^[^ ^24^ ^]^ These results provide evidence of the successful formation of salphen‐metal units. The peaks of 3D‐NiSAs/NiNCs‐POPs in its N 1s and O 1s spectra shifted to higher binding energy as compared to those of 3D‐NiSAs‐POPs, suggesting a interaction between Ni_n_ NCs and N, O atoms. The high‐resolution Ni 2p spectra of the samples contained three peaks at 856.3 eV, 855.3 eV, and 853.3 eV corresponding to Ni(II), Ni(I), and Ni(0), respectively, indicating the valence state of Ni between Ni^0^ and Ni^2+^.^[^ ^25^ ^]^

The coordinate geometry and local structure of Ni species in 3D‐NiSAs/NiNCs‐POPs and 3D‐NiSAs‐POPs were recorded by X‐ray absorption spectroscopy (XAS). Ni foil, NiO, and nickel (II) phthalocyanine (NiPc) were used as references. In the Ni K‐edge X‐ray absorption near‐edge structure (XANES) profiles shown in Figure 2c, the near‐edge absorption of 3D‐NiSAs‐POPs was close to that of NiO, suggesting that the valence state of Ni SAs was nearly +2. The near‐edge absorption of 3D‐NiSAs/NiNCs‐POPs was close to that of the Ni foil, implying the valence state of Ni atoms was lower than that of 3D‐NiSAs‐POPs due to the formation of Ni clusters. The Fourier‐transformed (FT) Ni K‐edge extended X‐ray absorption fine structures (EXAFS) spectra shown in Figure 2d reveal that 3D‐NiSAs/NiNCs‐POPs had one primary peak corresponding to the Ni−Ni scattering path at 2.2 Å and a shoulder peak corresponding to the Ni−N/O scattering path at around 1.6 Å.^[^ ^25^, ^26^ ^]^ These results further demonstrate the co‐existence of Ni_1_ SAs and Ni_n_ NCs in 3D‐NiSAs/NiNCs‐POPs. The 3D‐NiSAs‐POPs structure had a dominant peak corresponding to Ni−N/O coordination bonds at 1.6 Å. No Ni−Ni bonds at 2.2 Å were detected, confirming the non‐existence of Ni clusters. The EXAFS fitting analysis of the samples was further executed (Figures 2e,f; Figure S12 (Supporting Information)). The average coordination numbers (CN) of Ni−O, Ni−N, and Ni−Ni in 3D‐NiSAs/NiNCs‐POPs were 1.8, 1.4, and 4.7, respectively, and their average bond lengths were around 2.1 Å, 2.0 Å, and 2.5 Å, respectively (Table S2, Supporting Information). The average CNs of Ni−O and Ni−N in 3D‐NiSAs‐POPs were 2.3 and 2.2, respectively, and their average bond lengths were around 2.1 Å and 2.0 Å, respectively. The contour plots obtained from the wavelet transform (WT) EXAFS analysis show the coexistence of Ni‐N/O sites and Ni NCs in 3D‐NiSAs/NiNCs‐POPs (Figure 2g).

The permanent porosity of the samples was evaluated by N_2_ adsorption measurements at 77 K (Figure 2b). The 3D‐NiSAs/NiNCs‐POPs structure exhibited a typical type I isotherm with a Brunauer‐Emmett‐Teller (BET) surface area of 710 m^2^ g^−1^. The 3D‐POPs and 3D‐NiSACs‐POPs samples had relatively lower surface areas of 392 m^2^ g^−1^ and 262 m^2^ g^−1^, respectively. The considerably higher surface area of 3D‐NiSAs/NiNCs‐POPs might result from the metal ion source incorporated during the polymerization process is more favorable for producing metal‐salphen pocket.^[^ ^14^, ^27^ ^]^ The pore size distribution analysis based on the non‐local density functional theory (NLDFT) asserts that 3D‐NiSAs/NiNCs‐POPs possess micro‐pores with a pore diameter of 0.64 nm (Figure S13, Supporting Information). Hence, 3D‐NiSAs/NiNCs‐POPs with a high surface area and abundant exposed active sites is highly favorable for electron and mass transfer.

The optical absorption capacities and band structures of the 3D POPs based catalysts were investigated by solid‐state UV–vis diffuse reflectance spectra (UV–vis DRS) and Mott‐Schottky plots. It is noticeable from Figure S14 (Supporting Information) that 3D‐NiSAs/NiNCs‐POPs experienced a red shift in its optical adsorption edge than that of 3D‐NiSAs‐POPs, implying that the incorporation of metal clusters contributed to the visible light absorption capacity of 3D‐NiSAs/NiNCs‐POPs, and it can be ascribed to the increased delocalization with the chelation of metal clusters.^[^ ^28^ ^]^ The optical bandgaps of 3D‐NiSAs/NiNCs‐POPs, 3D‐NiSAs‐POPs, and 3D‐POPs were calculated from their Tauc‐plots as 1.68 eV, 1.94 eV, and 2.22 eV, respectively (Figure S15, Supporting Information). The Mott‐Schottky curves in Figures S16a−c (Supporting Information) confirm that all samples were n‐type semiconductors with typical positive slopes of C^−2^ values. The conduction band minimum (CBM) potentials of 3D‐NiSAs/NiNCs‐POPs, 3D‐NiSAs‐POPs, and 3D‐POPs were determined as −0.77 V, −0.80 V, and −0.81 V (versus the normal hydrogen electrode (NHE)), respectively, and their corresponding valence band maximum (VBM) potentials were 0.91 V, 1.14 V, and 1.41 V respectively (Figure S16d, Supporting Information). As illustrated in Figure S17 (Supporting Information), the CBM values of all materials were negative with respect to the redox potential of the CO_2_ to CO photoreduction process (−0.53 V vs NHE) and were positive with respect to the lowest unoccupied molecular orbital energy level of Ru(bpy)_3_Cl_2_·6H_2_O (−1.31 V vs NHE). Thus, the photoinduced electrons transferred from photosensitizer to catalysts, and the photocatalytic CO_2_RR was thermodynamic feasible.

**Figure 3:**
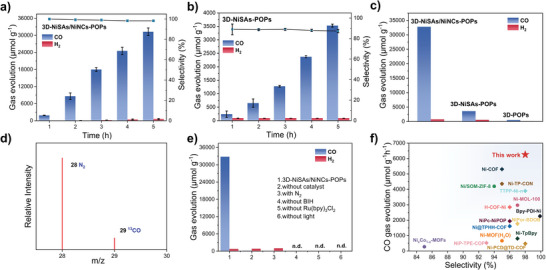
a, b) CO and H_2_ evolution with irradiation time during the photocatalytic CO_2_ reduction catalyzed by 3D‐NiSAs/NiNCs‐POPs and 3D‐NiSAs‐POPs under visible light irradiation. c) Comparison of the photocatalytic activity of 3D‐NiSAs/NiNCs‐POPs, 3D‐NiSAs‐POPs and 3D‐POPs. d) Gas chromatography‐mass spectrometry for ^13^C isotope tracer tests of 3D‐NiSAs/NiNCs‐POPs. e) Control experiments of the photocatalytic CO_2_ reduction performance under different altered conditions. f) Comparison of the CO production rate and selectivity of 3D‐NiSAs/NiNCs‐POPs with other reported Ni‐based porous photocatalysts.

Uniformly dispersed metal active sites, well‐developed pore structure and intriguing photophysical property endowed the 3D‐NiSAs/NiNCs‐POPs a potential to be a fascinating photocatalyst for CO_2_RR. The photocatalytic CO_2_RR of the as‐prepared catalysts were carried out by a simulated visible light (λ > 420 nm)‐driven system in a CO_2_‐saturated solution consisting of acetonitrile and deionized water. In this experiment, Ru(bpy)_3_Cl_2_·6H_2_O and 1,3‐dimethyl‐2‐phenyl‐2,3‐dihydro‐1H‐benzo[d]imidazole (BIH) were used as the photosensitizer and the hole scavenger, respectively. The effects of the acetonitrile/H_2_O ratio and the catalyst dosage on the yield and selectivity of CO were explored using a white LED lamp as the light source to screen the best photocatalytic conditions. The optimum yield and selectivity of CO were achieved when the acetonitrile/H_2_O ratio was 3:2 and the catalyst dosage was 1 mg (Figure S18, Supporting Information). Under the optimized conditions, the CO_2_ to CO photoreduction reaction was performed using a 300 W Xe lamp as the light source. In our photocatalytic system, CO and H_2_ were identified as the main products by gas chromatography and ^1^H NMR (Figure S19, Supporting Information). It is observable from **Figure** **3**a that CO was continuously produced under visible light irradiation for five hours; however, the production rate decreased after two hours under light illumination, and it can be attributed to the degradation of the photosensitizer (Ru(bpy)_3_Cl_2_·6H_2_O).^[^ ^29^ ^]^ The CO production in 3D‐NiSAs/NiNCs‐POPs reached 31 200 µmol g^−1^ with a remarkable selectivity of 98% after five hours of light irradiation, and these values are superior to most Ni‐based porous photocatalysts (Figure 3f; Table S4, Supporting Information). The control sample of 3D‐NiSAs‐POPs had a CO production of 3530 µmol g^−1^ and an H_2_ production of 546 µmol g^−1^ under identical catalytic conditions (Selectivity = 87%) (Figure 3b), and these values were 8.8 times lower than those of 3D‐NiSAs/NiNCs‐POPs with the same Ni loading (Figure 3c). Only a small amount of CO (540 µmol g^−1^) was generated in pure 3D‐POPs due to the lack of metal catalytic sites. These results confirm that both NCs and SAs contributed to the high CO_2_ reduction performance of 3D‐NiSAs/NiNCs‐POPs. The 3D framework played significant roles in stabilizing metal sites (SAs and NCs) in the catalytic process and facilitating electron transfer. A mixed gas containing 15% CO_2_ and 85% N_2_ was used to evaluated the conversion of post‐combustion CO_2_. When the reaction was performed under the same conditions for 5 h, 10 996 µmol g^−1^ of CO with 74% selectivity over H_2_ was obtained (Figure S20, Supporting information). The apparent quantum yields (AQE) of 3D‐NiSAs/NiNCs‐POPs for CO production was measured with different monochromatic light. The highest value was 0.10% at 450 nm (Figure S21, Supporting information).

To determine the carbon origin of photoreduced products, a ^13^C isotope labeling CO_2_ experiment on 3D‐NiSAs/NiNCs‐POPs was conducted (Figure 3d). A peak of ^13^CO (m/z = 29) was detected by mass spectrometry, indicating that CO was generated from reactant CO_2_ rather than other carbon sources. Furthermore, the replacement of CO_2_ with N_2_ resulted in the detection of only H_2_, confirming that CO originated from CO_2_ (Figure 3e). The control experiment of removing Ru(bpy)_3_Cl_2_·6H_2_O from the system revealed that the reaction was light‐driven and photogenerated electrons were produced from the Ru photosensitizer. Moreover, no apparently reduction products could be detected when the BIH is absence, as it consumes the photogenerated holes and inhibited the recombination of photogenerated carriers. In addition, to examine the catalytic stability of 3D‐NiSAs/NiNCs‐POPs, fresh Ru(bpy)_3_Cl_2_·6H_2_O and BIH were added to the system after each cycle to maintain a continuous process. After three successive cycles test of catalyst durability, there is no apparent attenuation in CO production, but the selectivity has a slight deterioration (Figure S22, Supporting Information). The structural stability of post‐catalytic 3D‐NiSAs/NiNCs‐POPs was characterized by XPS and FT‐IR, and no Ni cluster agglomeration occurred after the photocatalytic reduction. (Figure S23, Supporting Information).

Photoelectrochemical measurements were performed to elucidate the separation and transfer capability of photogenerated carriers in 3D‐NiSAs/NiNCs‐POPs. It can be inferred from the electrochemical impedance spectroscopy (EIS) results in Figure S24 (Supporting Information) that 3D‐NiSAs/NiNCs‐POPs had a higher charge immigration efficiency than 3D‐POPs and 3D‐NiSAs‐POPs. The increased transient photocurrent density of 3D‐NiSAs/NiNCs‐POPs further confirms that the construction of the NC‐AC‐based catalysts favored the charge transfer (Figure S25, Supporting Information). Moreover, the steady‐state photoluminescence (PL) spectra quenching experiments indicated that the strong emission of the photosensitizer (Ru(bpy)_3_Cl_2_·6H_2_O) at 625 nm was continuously attenuated with the addition of the catalysts (Figure S26, Supporting Information). The markedly more attenuated intensity of 3D‐NiSAs/NiNCs‐POPs than that of 3D‐NiSAs‐POPs is attributed to the improved exciton splitting and photoexcited electron transfer. The time‐resolved photoluminescence measurements in Figure S27 (Supporting Information) illustrate that the average lifetime of photogenerated charges of Ru(bpy)_3_Cl_2_·6H_2_O decreased in the presence of 3D‐NiSAs/NiNCs‐POPs, demonstrating that the recombination of photogenerated electrons and holes was inhibited strongly after the loading of Ni SAs and NCs. A quenching effect was also observed when BIH was added to the Ru solution, which evidenced photogenerated holes were compensated by BIH to reach the charge balance (Figure S28, Supporting Information).

**Figure 4:**
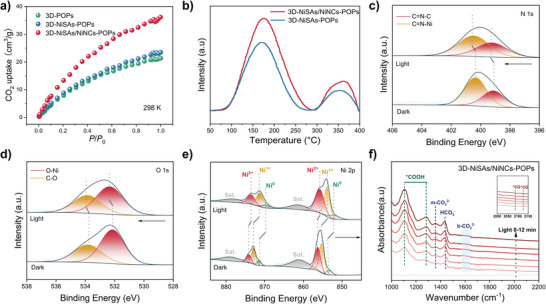
a) CO_2_ adsorption isotherms of 3D‐NiSAs/NiNCs‐POPs, 3D‐POPs and 3D‐NiSAs‐POPs at 298 K. b) CO_2_‐TPD spectra of 3D‐NiSAs/NiNCs‐POPs and 3D‐NiSAs‐POPs. c, d, e) In situ XPS spectra of O 1s, N 1s and Ni 2p in 3D‐NiSAs/NiNCs‐POPs under irradiation. f) In situ DRIFTS spectra of 3D‐NiSAs/NiNCs‐POPs.

To gain a deep understanding of the mechanism of photocatalytic CO_2_ reduction, the affinity between CO_2_ and the catalysts was explored. The CO_2_ adsorption isotherms shown in **Figures** **4**a and S29 (Supporting Information) indicate that the CO_2_ capacity of 3D‐NiSAs/NCs‐POPs (43 cm^3^ g^−1^ at 273 K, 36 cm^3^ g^−1^ at 298 K) was higher than 3D‐NiSAs‐POPs (27 cm^3^ g^−1^ at 273 K, 23 cm^3^ g^−1^ at 298 K). Based on the isotherms at 273 K and 298 K, the isosteric heat of adsorption (Q_st_) for 3D‐NiSAs/NiNCs‐POPs, 3D‐NiSAs‐POPs, and 3D‐POPs was calculated (24.27 kJ mol^−1^, 18.55 kJ mol^−1^ and 15.65 kJ mol^−1^). These results assert that the increased Ni sites in 3D‐NiSAs/NiNCs‐POPs greatly enhanced the CO_2_ affinity, and it can be attributed to Lewis acid‐base interactions between adsorbed CO_2_ molecules and Ni atoms. Moreover, the surface adsorption properties of the catalysts were investigated by a CO_2_‐temperature programmed desorption (CO_2_‐TPD) test (Figure 4b). Two major peaks at 170 °C (weak basic site desorption peak) and 350 °C (medium basic site desorption peak) were observed.^[^ ^30^ ^]^ In the case of 3D‐NiSAs/NiNCs‐POPs, the higher integrating peak area and its slight shift toward the high‐temperature region indicate an enhanced amount of basic sites and a stronger CO_2_ adsorption force. These results strongly prove that the introduction of Ni_n_ NCs induced a large amount of CO_2_ adsorption sites in 3D‐NiSAs/NiNCs‐POPs.

For further understand the possible catalytic mechanism, quasi in situ XPS was performed to detect the flow direction of photoelectron migration. After light irradiation, the binding energy of N 1s, O 1s, and C 1s in 3D‐NiSAs/NiNCs‐POPs shifted positively, whereas that of Ni 2p shifted negatively, illustrating the electron transfer from the POPs to Ni sites (Figure 4c−e; Figure S30, Supporting Information). To determine reaction intermediates and the possible reaction pathway, in situ diffuse reflectance infrared Fourier transforms spectroscopy (DRIFTS) was employed (Figure 4f; Figure S31, Supporting Information). The peaks at 1113 cm^−1^ and 1265 cm^−1^ appeared from *COOH, which was a critical intermediate during the CO_2_ to CO photoreduction reaction.^[^ ^9^, ^31^ ^]^ The presence of bicarbonate species (HCO_3_^−^), monodentate carbonate (m−CO_3_^2−^) and bidentate carbonate (b−CO_3_^2−^) species can be elucidated according to the absorption bands of 1432 cm^−1^, 1352 cm^−1^ and 1598 cm^−1^ were observed during photoreduction CO_2_ to CO.^[^ ^9^, ^31^, ^32^ ^]^ The intensities of these characteristic absorption bands gradually increased with the prolongation of irradiation time. In addition, the two peaks at 2087 cm^−1^ and 2128 cm^−1^ originated from the *CO intermediate in the photocatalytic process.^[^ ^33^ ^]^ The in situ FTIR analysis confirms that *COOH and *CO intermediates were involved in the CO_2_ to CO photocatalytic process.

**Figure 5:**
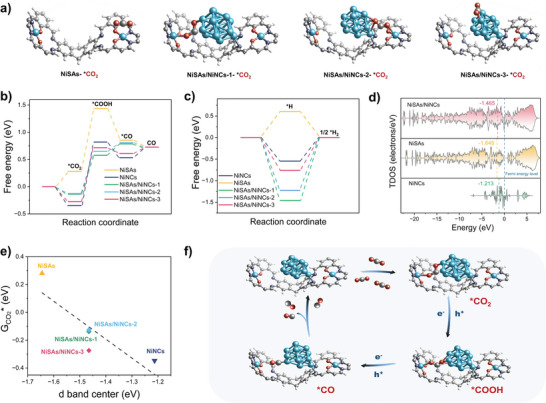
a) Adsorption configurations of CO_2_ involved in the computational models of NiSAs, NiSAs/NiNCs‐1, NiSAs/NiNCs‐2 and NiSAs/NiNCs‐3 (Blue, purple, grey, red, and white balls represent Ni, N, C, O, and H, respectively). Calculated free energy diagram of b) the CO_2_RR to CO photocatalytic reaction and c) HER. d) Calculated total density of state of NiNCs, NiSAs, and NiSAs/NiNCs. e) Scaling relationship between the d‐band centers of different computational catalyst models and the adsorption free energy of *CO_2_. f) Proposed CO_2_ to CO photocatalytic pathway in the model of NiSAs/NiNCs‐1.

DFT calculations were carried out to explore the synergistic effect of SAs and NCs on CO_2_ photocatalytic reduction. The initial adsorption of CO_2_ occurred through the coordination of Ni sites with O atoms and generated intermediate *CO_2_. Three possible NC‐SA models were considered (**Figure** **5**a). In the first NC‐SA model, a Ni SA and a NC anchoring to the neighboring salphen unit as active sites were bonded with two O atoms of CO_2_ (denoted as NiSAs/NiNCs‐1‐*CO_2_). In the second model, a Ni SA and a NC were located at the same salphen unit and got bonded with CO_2_ to produce a transition state of NiSAs/NiNCs‐2‐*CO_2_. In the third model, a Ni SA and a NC were located at the same salphen unit; however, only surface Ni atoms in the NC adsorbed CO_2_ to produce NiSAs/NiNCs‐3‐*CO_2_. Notably, after the adsorption of CO_2_ molecules on the catalyst model of NiSAs/NiNCs‐1 and NiSAs/NiNCs‐2, the O═C═O bond angle was reduced from 180° to 115° and 128°, respectively, whereas the C═O bond length was extended from 1.16 Å to 1.61 Å and 1.22 Å and the other C═O bond length was extended to 1.52 Å and 1.35 Å, respectively (Table S3, Supporting Information). The asymmetrical interaction between the SA and the NC favored the bending of CO_2_ molecules and increased the dipole, thus creating a lower barrier for electron acceptance. In all three models, this stage was exothermic, indicating that CO_2_ can be adsorbed spontaneously and these coordination modes stabilizing the intermediates (Figure 5b). Considering the similar energy barrier of the NiSAs/NiNCs‐1 and NiSAs/NiNCs‐2 models, CO_2_ might be adsorbed and activated by a variety of sites in the 3D framework. When the SAs counterparts adsorbed CO_2_, the formation of NiSAs‐*CO_2_ was an endothermic process and involved a stronger activation energy barrier. These results imply that the co‐existence of SAs and NCs significantly promoted the adsorption and activation of CO_2_ and facilitated subsequent reduction reactions.

With the continuation of the reaction with photogenerated electrons, protons were transferred to *CO_2_ to generate *COOH. This was a rate‐determining step, and the free energy barriers for SAs, isolated NCs, NiSAs/NiNCs‐1, NiSAs/NiNCs‐2 and NiSAs/NiNCs‐3 were 1.16 eV, 1.17 eV, 0.71 eV, 0.77 eV, and 0.99 eV, respectively. The lower energy barrier of the NC‐SA hybrid catalysts suggests that the co‐contribution of SAs and NCs as active sites more thermodynamically favored the photoreduction than the isolated SAs and NCs. Among them, NiSAs/NiNCs‐1 was the optimal one because its microenvironment facilitated the addition of protons to form *COOH (key immediate). Subsequently, *CO was produced by another hydrogen‐assisted electron reduction process. Finally, *CO left the Ni site and returned to the original state of the photocatalyst (Figure 5f; Figures S32−S35, Supporting Information). In NiSAs, NiSAs/NiNCs‐1, and NiSAs/NiNCs‐2, the desorption of *CO released 0.13 eV, 0.06 eV, and 0.08 eV of energy, respectively, whereas for NiNCs and NiSAs/NiNCs‐3, the desorption of *CO required 0.19 eV and 0.12 eV of energy expenditure, respectively. A comparison of all pathways along with the Gibbs free energy diagram is presented in Figure 5b. Clearly, the co‐contribution of Ni SAs and NCs lowered the overall activation energy barrier of the CO formation process, and greatly increased the reaction activity. Further, the Gibbs free energy changes of these models during the competitive side hydrogen evolution reaction (HER) over these structures were calculated (Figure 5c). The Gibbs free energy changes of NiSAs, NiNCs and NiSAs/NiNCs‐1/2/3 *H formation were calculated as 0.60 eV, 0.55 eV, 1.46 eV, 1,22 eV, and 0.76 eV, respectively, indicating that NiSAs/NiNCs‐1 had the weakest binding ability for *H species. These results reveal that the synergistic effect of Ni SAs and NCs inhibited the HER reaction, leading to high CO selectivity.

The density of state (DOS) diagrams in Figure 5d illustrate that the d‐band centers of NiSAs/NiNCs, NiSAs, and NiNCs. It was found that the interaction between Ni−N_2_O_2_ and Ni clusters leads to a moderate d‐band center (Figure 5e). According to the d‐band theory, a moderate Fermi level induces a moderate substrate adsorption ability for catalyst.^[^ ^34^ ^]^ The strength of substrate adsorption on catalysts determines their catalytic activity. Moderate strength is favorable to catalytic reactions, whereas too strong or too weak strength reduces the catalytic ability of catalysts.^[^ ^8a^ ^]^ The relationship between the d‐band center position of these models and their CO_2_ activation energy barrier indicates that the synergistic effect of Ni SAs and NCs modulated the adsorption energy, reduced the activation energy, and enhanced the activation of CO_2_ on the catalysts.

Based on the above experimental and theoretical results, a possible mechanism was proposed for CO_2_ photoreduction catalyzed by 3D‐NiSAs/NiNCs‐POPs (Figure S36, Supporting Information). Initially, CO_2_ molecules were adsorbed on the catalyst through asymmetric bonding with two O atoms. Under visible light irradiation, the photosensitizer (Ru(bpy)_3_Cl_2_·6H_2_O) became excited, and induced electrons were transferred to Ni sites of the 3D matrix. The remaining holes were captured by the electron donor (BIH), and the entire cycle was closed. The synergistic interaction between single Ni atoms and Ni clusters in 3D‐NiSAs/NiNCs‐POPs promoted the activation of CO_2_, modulated the bonding strength with intermediates, lowered the energy barrier, and effectively boosted the CO_2_ to CO photocatalytic reaction.

## 3 Conclusion

In conclusion, we developed a synergic Ni NC‐SA based 3D porous framework (3D‐NiSAs/NiNCs‐POPs) as an active CO_2_RR catalyst through a one‐step synthesis route. 3D‐NiSAs/NiNCs‐POPs showed the high activity towards CO production from CO_2_ photoreduction (CO production rates of 6.24 mmol g^−1^ h^−1^, selectivity of 98%), suppressing the SA counterpart (3D‐NiSAs‐POPs). Spectroscopic, catalytic data and theoretical calculations results suggest that the exceptional activity of 3D‐NiSAs/NiNCs‐POPs originates from the cooperation of SAs and NCs activated CO_2_ molecules, decreased the activation energy barrier, accelerated the reaction kinetics, and optimized the binding strength with intermediates. This work provides new insights into the adsorption and activation of CO_2_ molecules for NC‐SA catalyst in 3D porous framework, it should be helpful toward developing other photocatalysts from reticular nanomaterials.

## 4 Experimental Section

The Experimental Section was available in the Supporting Information.

## Conflict of Interest

The authors declare no conflict of interest.

## Supporting information

Supporting Information

## Data Availability

Research data are not shared.
