# Supplementary material for: Asymmetrical Interactions between Ni Single Atomic Sites and Ni Clusters in a 3D Porous Organic Framework for Enhanced CO2 Photoreduction
Source: Adv Sci (Weinh). 2024 Mar 15;11(23):2401508. doi: 10.1002/advs.202401508 (PMC11187926; doi:10.1002/advs.202401508)
Supplement: Supplementary file 1 — Supporting Information [file ADVS-11-2401508-s001.pdf]

ADVANCED SCIENCE
Open Access

Supporting Information

for *Adv. Sci.*, DOI 10.1002/advs.202401508

Asymmetrical Interactions between Ni Single Atomic Sites and Ni Clusters in a 3D Porous Organic Framework for Enhanced CO_2 Photoreduction

Fang-Qin Yan, Xiao-Yu Dong, Yi-Man Wang, Qian-You Wang, Shan Wang and Shuang-Quan Zang**

Supporting Information

Asymmetrical Interactions between Ni Single Atomic Sites and Ni Clusters in a Three-Dimensional Porous Organic Framework for Enhanced CO_2 Photoreduction

Fang-Qin Yan[+] Xiao-Yu Dong[+], Yi-Man Wang, Qian-You Wang, Shan Wang & Shuang-Quan Zang**

Henan Key Laboratory of Crystalline Molecular Functional Materials, and College of Chemistry, Zhengzhou University, Zhengzhou, 450001, China.

E-mail: zangsqzg@zzu.edu.cn; shanwang@zzu.edu.cn

[+] These authors contributed equally to this work.

Materials characterization.

Powder X-ray diffraction (PXRD) was collected on a Riguku D / Max-2500PC X-ray diffractometer with Cu sealed tub (λ = 1.54178 Å).

Fourier transform infrared (FT-IR) spectra were recorded on a Bruker ALPHA II FT-IR spectrometer.

The X-ray photoelectron spectroscopy (XPS) measurements were tested using Thermo Scientific K-Alpha. The C peak at 284.8 eV was used as a reference to correct for charging effects. In situ XPS (Thermo Fischer, ESCALAB 250Xi) was conducted by Thermo Fisher Scientific (ESCALAB 250Xi) equipped with light source device.

Solid-state UV-vis diffuse reflectance spectra were collected on UH4150 spectrophotometer using $BaSO_4$ as the reference.

Nitrogen sorption isotherms were measured at liquid nitrogen temperature (77 K) by using automatic volumetric adsorption equipment (Belsorp Max) after a degassed process at 120 ℃ for 12 h. Specific surface areas were obtained by using the Brunauer-Emmet-Teller (BET) model, pore size distributions were simulated by the nonlocal density functional theory (NLDFT) model.

CO_2 adsorption isotherms were measured at 273 K and 298 K by using automatic volumetric adsorption equipment (Belsorp Max) after a degassed process at 120 ℃ for 12 h.

The inductively coupled plasma mass spectrometry (ICP-MS) analysis was recorded on Agilent 5110 (OES).

Morphology of all samples were carried out using Zeiss Sigma 500 on a scanning electron microscopy (SEM) measurement.

Transmission electron microscopy (TEM) images were obtained in FEI TalosF200S.

Atomic-resolution high-angle annular dark-field scanning transmission electron microscopy (HAADF-STEM) images were obtained in FEI Titan cubed Themis G2 300 STEM with aspherical aberration corrector.

Photoluminescence spectra and luminescence decay was measured on a HORIBA FluoroLog-3 fluorescence spectrometer.

Electrochemical test was carried out on electrochemical working station CHI 660E (Shanghai).

Isotopic labeling control experiments were obtained on gas chromatography-mass spectrometry (7890A and 5975C, Agilent Technologies).

Thermogravimetric analysis (TGA) was recorded by a TGA Q50 thermal analyzer from room temperature to 800 °C under N_2 atmosphere using a heating rate of 10 °C/min.

The produced gas was monitored by Agilent GC7820 Gas Chromatograph (N_2 as gas carrier, and the columns of GC are Porapak Q and MolSieve 5A).

The CO_2RR reaction pathways of as-prepared catalysts were detected via in situ DRIFTS spectrometer (BRUKER INVENIO S).
Xianquan TP5058 (TianJin, China) was used to conduct CO_2 temperature programmed desorption (CO_2-TPD) on materials.

Experimental Section

Unless otherwise specified, all chemicals were obtained from commercial source without additional drying or degassing. 3,3',5,5'-tetraformyl-4,4'-biphenyldiol denoted as TFBD. 2,3,6,7,14,15-Hexaaminotriptycene hexahydro chloride denoted as HATT.

Synthesis of 3D-NiSAs/NiNCs-POPs: In a 25 mL Schlenk flask, HATT (67.6 mg, 0.12 mmol,1 equiv.), TFBD (53.6 mg, 0.18 mmol, 1.5 equiv.) and $Ni(OAc)_2{\cdot}4H_2O$ (98.4 mg, 0.396 mmol, 3.3 equiv.) were mixed with 10 mL dry DMF. The mixture was sonicated few minutes until it becomes homogeneous solution. After stirring and refluxing at 100 °C for 16 h, the suspension was cooled to room temperature. The precipitate was separated by filtration and washed with DMF (3 ×10 mL), MeOH (3 ×10 mL) as well as Soxhlet extractor washing with THF for 24 hours for further purification. Finally, the reddish brown solid was dried at 80 °C under vacuum overnight to obtain 120 mg of **3D-NiSAs/NiNCs-POPs** with a yield of 55%.

Synthesis of 3D-POPs: HATT (16.9 mg, 0.03 mmol,1 equiv.), TFBD (13.4 mg, 0.045 mmol, 1.5 equiv.) were carefully placed into a vial with a mixed solution of DMF (0.2 mL) and n-BuOH (0.8 mL). The mixture was sonicated few minutes until it becomes homogeneous solution, then aqueous acetic acid (6.0 M, 0.2 mL) was added. After heating in an isothermal oven at 120℃ for 5 days, the mixture was cooled to room temperature. The resulting brown solid was soaked in DMF solvent for 3 days (the solvent was changed many times during this period) to remove all unreacted TFBD and HATT. After this process, the sampled was isolated and activated by Soxhlet extractor with THF for 2 days and dried at 80 ℃ under vacuum overnight to give **3D-POPs** in a yield of 50%.

Synthesis of 3D-NiSAs-POPs: 3D-POPs (40 mg) was added in the solution of Ni $(OAc)_2 \cdot 4H_2O$ (160 mg) in MeOH (80 mL) at room temperature, then the suspension was stirred for 3 days. The precipitate was isolated after centrifuged, washed with MeOH for 12 h to ensure complete removal of residual Ni $(OAc)_2 \cdot 4H_2O$, and then dried at 80 ℃ under vacuum overnight, to give **3D-NiSAs-POPs**.

Photocatalytic Experiments: The photocatalytic CO_2 reduction experiments of all the synthesized catalyst was carried out in a 50 mL double-walled quartz reactor with a 300 W Xenon lamp equipped with UV cutoff filter ($\lambda > 420$ nm) as light source. The reaction was kept at ambient temperature for stirring, the detailed procedure is as follows: Primarily, 1 mg catalyst, 5 mg photosensitizer ($Ru(bpy)_3Cl_2 \cdot 6H_2O$) and 25 mg sacrificial agent (1,3-dimethyl-2-phenyl-2,3-dihydro-1H-benzo[d]imidazole, BIH) were put into a mixed solvent system of 4 mL deionized water and 6 mL acetonitrile. After sonicating, the suspension become homogeneous. Next, high-purity carbon dioxide was introduced into the reaction system for 25 min to remove the air and full of CO_2 atmosphere. The straight-line distance from light source to the outer wall of the reactor was fixed to 3 cm. Agilent GC7820 Gas Chromatograph equipped with FID detector and TCD detector was used to monitor the gas product (300 μL of the headspace was injected into the gas chromatography every hour.). Three runs of consecutive photocatalytic reduction of CO_2 to CO by adding equal amounts of $Ru(bpy)_3Cl_2 \cdot 6H_2O$ and BIH at each run to

the photocatalytic system after 3 h of reaction and reintroducing CO_2. In the pre-phase of photocatalytic experiment, using a white LED lamp (400 nm ≤ λ ≤ 800 nm) as the light source to screen the best photocatalytic conditions.

Photoelectrochemical measurements: All photoelectrochemical measurements (Electrochemical impedance spectroscopy, Mott-Schottky spots and Photocurrent) were performed on an electrochemical workstation CHI 660E (Chenhua Instrument, Shanghai, China) via a standard three-electrode system in 0.5 M Na_2SO_4 solution, which contains a working electrode, a platinum plate as counter electrode, and a saturated Ag/AgCl (KCl saturated) electrode as a reference electrode at ambient temperature. The catalyst (1 mg) was dispersed into a solution of 20 μL 5 wt% Nafion and 1 mL ethanol. For Mott-Schottky plot and electrochemical impedance spectroscopy (EIS) measurements, the resulting mixture was deposited onto the surface of ITO and left in the air for drying to prepare the working electrode. Mott-Schottky plots were recorded at frequencies of 500, 1000 and 1500 respectively. For photocurrent, glassy carbon electrode coated by $Ru(bpy)_3Cl_2\cdot 6H_2O$ and catalyst with Nafion as the working electrode and 300 W Xenon lamp with UV cutoff filter (λ > 400 nm) as light source.

In situ diffuse reflectance infrared Fourier transform spectroscopy (DRIFTS) measurements: In situ DRIFTS tests were implemented by Bruker INVENIO S FT-IR spectrophotometer equipped with an MCT detector cooled by liquid nitrogen and an in situ reaction cell. The detailed pretreatment and test conditions are given as follows. Firstly, the catalyst suspension was uniformly deposited onto the clean surface of Si prism and left in the air for drying. After that, carefully add 3-5 mL the same mixed solution as the photocatalytic (5 mg photosensitizer and 25 mg sacrificial agent in a mixed solvent system of 4 mL deionized water and 6 mL acetonitrile.) to the reaction cell and inject the high-purity CO_2 gas for 10 min until the solution was saturated. During the overall process, much attention should be paid to prevent the catalyst from falling off the Si prism. The background spectrum was collected before the Xenon light is turned on. Next, turn on the Xenon lamp and then the IR spectra were collected in situ through the MCT detector. Each spectrum was recorded by averaging 64 scans

at a 4 cm^{-1} spectral resolution.

CO_2-TPD measurement: The sample was dried and pre-treated at 10 ℃ min^{-1} from room temperature to 100 ℃ in a He stream (50 mL min^{-1}). When it is cooled to 50℃, 10% CO_2/He mixed gas was injected until adsorption saturation, and the He air flow is switched to 50mL min^{-1} to remove the physically adsorbed CO_2 on the surface. Then the thermal desorption of chemisorbed CO_2 was performed in flowing He at a ramp rate of 10 ℃ min^{-1} to a final temperature of 400 ℃. During the process, the outgoing gas was detected by TCD detector.

Apparent quantum efficiency: The apparent quantum efficiency (AQE) of the catalysts was measured by different bandpass filters (including 380, 420, 425, 475, 500 and 550 nm,) under the same photocatalytic reaction conditions. The AQE values were calculated as follow:

$$AQE(\%) = (2 \times n_{\mathrm{CO}} \times N_A \times h \times c) \times 100\%/(S \times P \times T \times \lambda)$$

where n_{CO} is the molar number of the CO, N_A is Avogadro's constant (6.022×10^{23} mol^{-1}), h is the Planck's constant (6.63×10^{-34} m^2 kg s^{-1}), c is the speed of light (3×10^8 m s^{-1}), S is the irradiation area (cm^2), P is irradiation intensity (W cm^{-2}), T is irradiation time (s), and λ is the wavelength of the light source, respectively.

Computational methods:

All the calculations were performed within the framework of the density functional theory (DFT) as implemented in the Vienna Ab initio Software Package (VASP 5.4.4) code within the Perdew–Burke–Ernzerhof (PBE) generalized gradient approximation and the projected augmented wave (PAW) method [1-4] . The cutoff energy for the plane-wave basis set was set to 450 eV. The Brillouin zone of the surface unit cell was sampled by Monkhorst–Pack (MP) grids, with k-point mesh density of $2\pi \times 0.04$ Å^{-1} for structures optimizations [5] . The convergence criterion for the electronic self-consistent iteration and force was set to 10^{-5} eV and 0.01 eV/Å, respectively. The vacuum layer of 15 Å was introduced to avoid interactions between periodic images.

The free energies of adsorbates at temperature T were estimated according to the harmonic approximation, and the entropy is evaluated using the following equation:

$$S(T) = K_B + \sum_{i}^{\text{harm DOF}} \left(\frac{\varepsilon_i}{K_B T(e^{\frac{\varepsilon_i}{K_B T}} - 1)} - \ln(1 - e^{-\frac{\varepsilon_i}{K_B T}}) \right)$$

where K_B is Boltzmann's constant and DOF is the number of harmonic energies (ε_i) used in the summation denoted as the degree of freedom, which is generally 3N, where N is the number of atoms in the adsorbates. Meanwhile, the free energies of gas phase species are corrected as:

$$G_g(T) = E_{elec} + E_{ZPE} + \int C_p \, dT - TS(T)$$

where C_p is the gas phase heat capacity as a function of temperature derived from Shomate equations and the corresponding parameters in the equations were obtained from NIST.

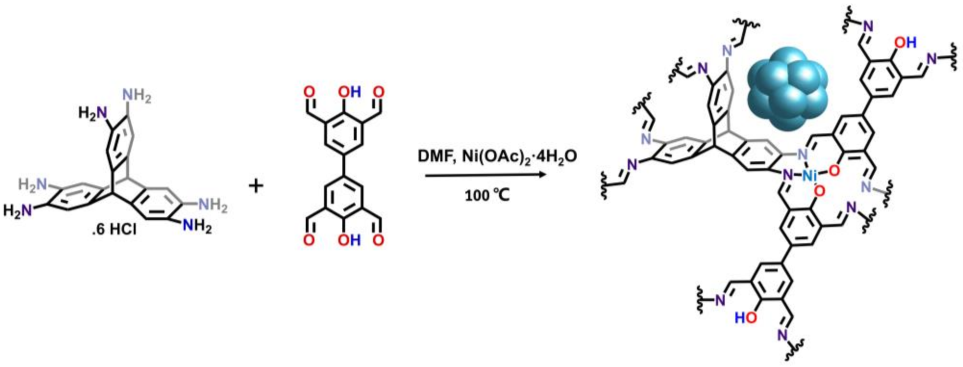

Figure S1. Schematic illustration route of the synthesis of 3D-NiSAs/NiNCs-POPs.

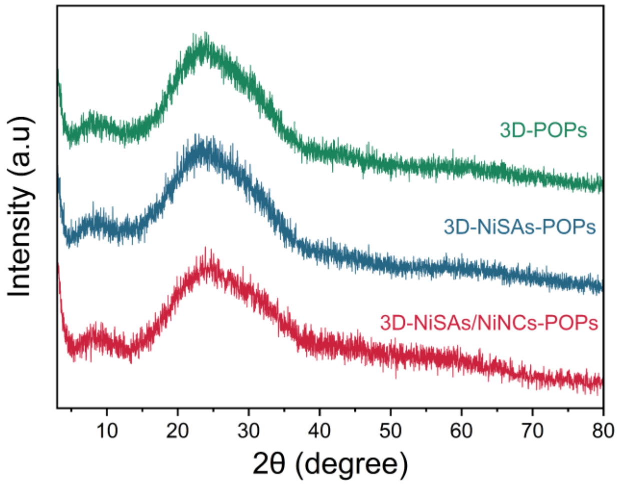

Figure S2. The PXRD patterns of 3D-NiSAs/NiNCs-POPs, 3D-NiSACs-POPs, 3D-POPs.

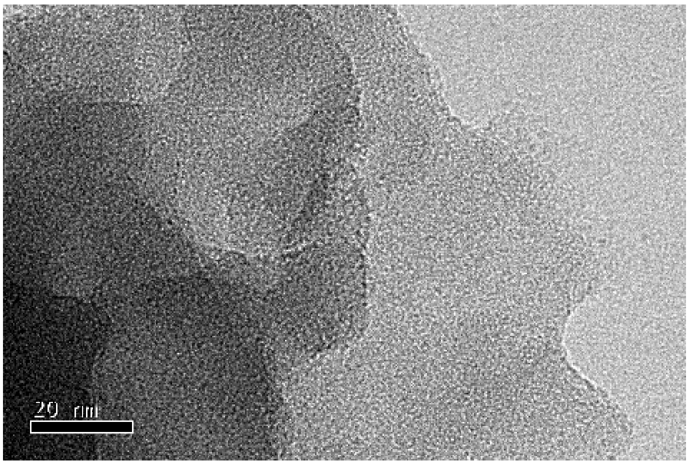

Figure S3. The TEM images of control experiments using1,4-dioxane as solvent.

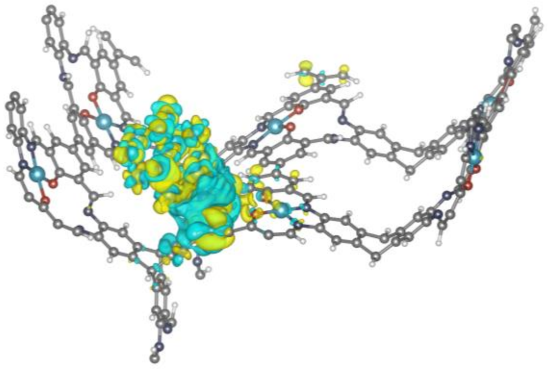

Figure S4. Differential charge density by representing the depletion (blue) and accumulation (yellow) of electron distributions.

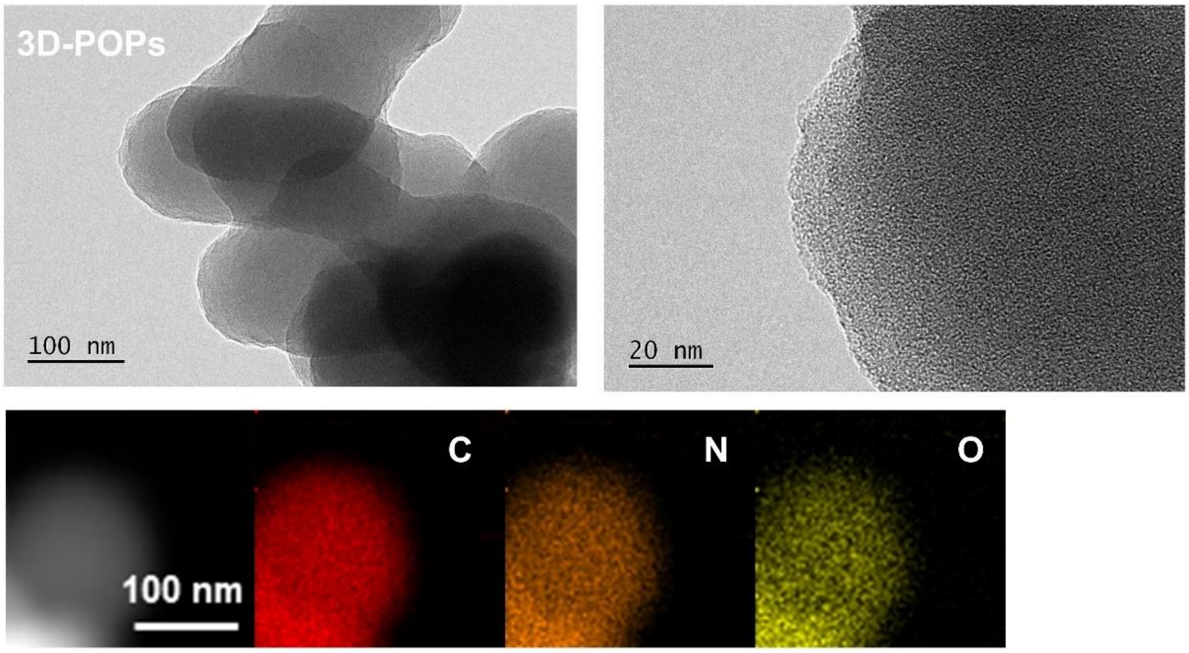

Figure S5. TEM images of 3D-POPs and corresponding EDS element mapping images.

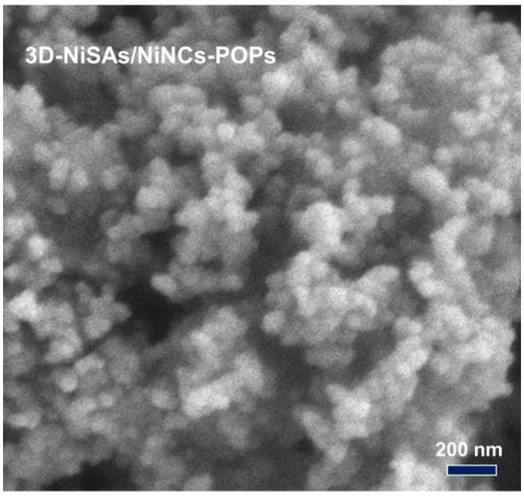

Figure S6. SEM images of 3D-NiSAs/NiNCs-POPs

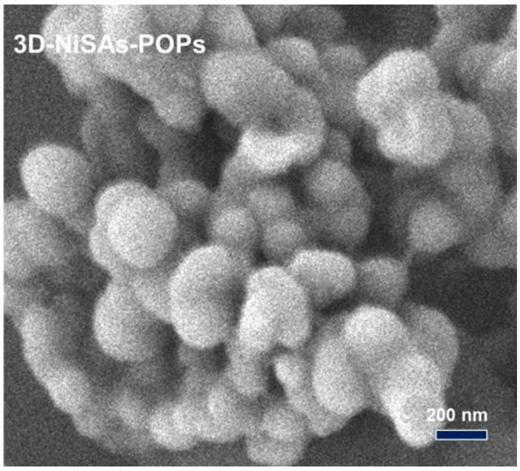

Figure S7. SEM images of 3D-NiSAs-POPs.

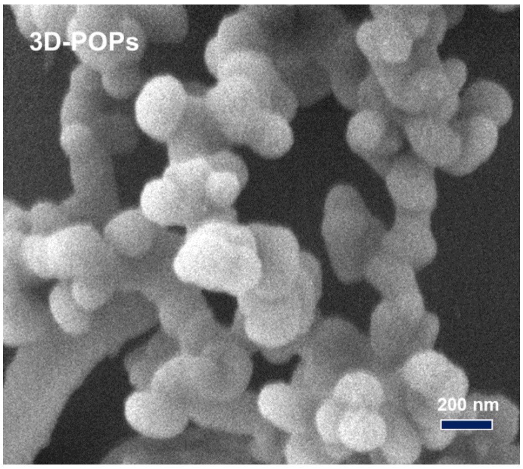

Figure S8. SEM images of 3D-POPs.

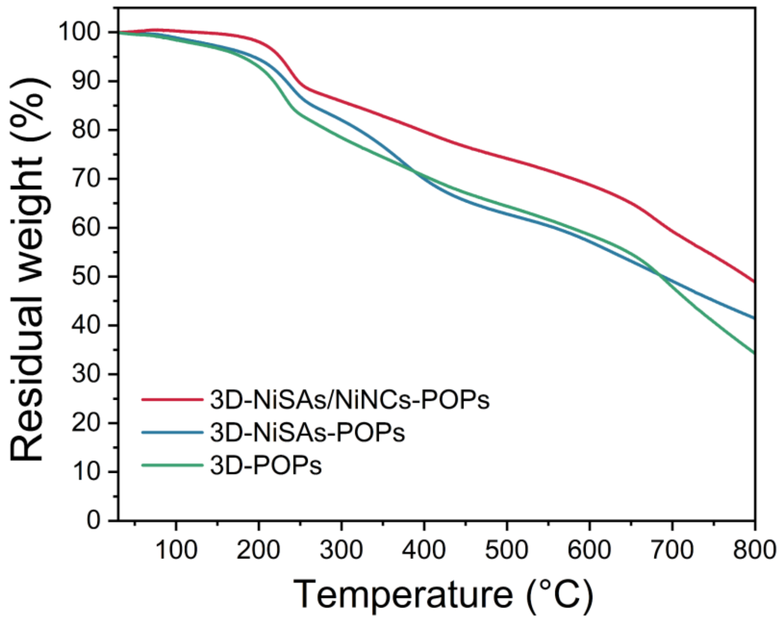

Figure S9. TGA curves of 3D-NiSAs/NiNCs-POPs, 3D-NiSAs-POPs and 3D-POPs.

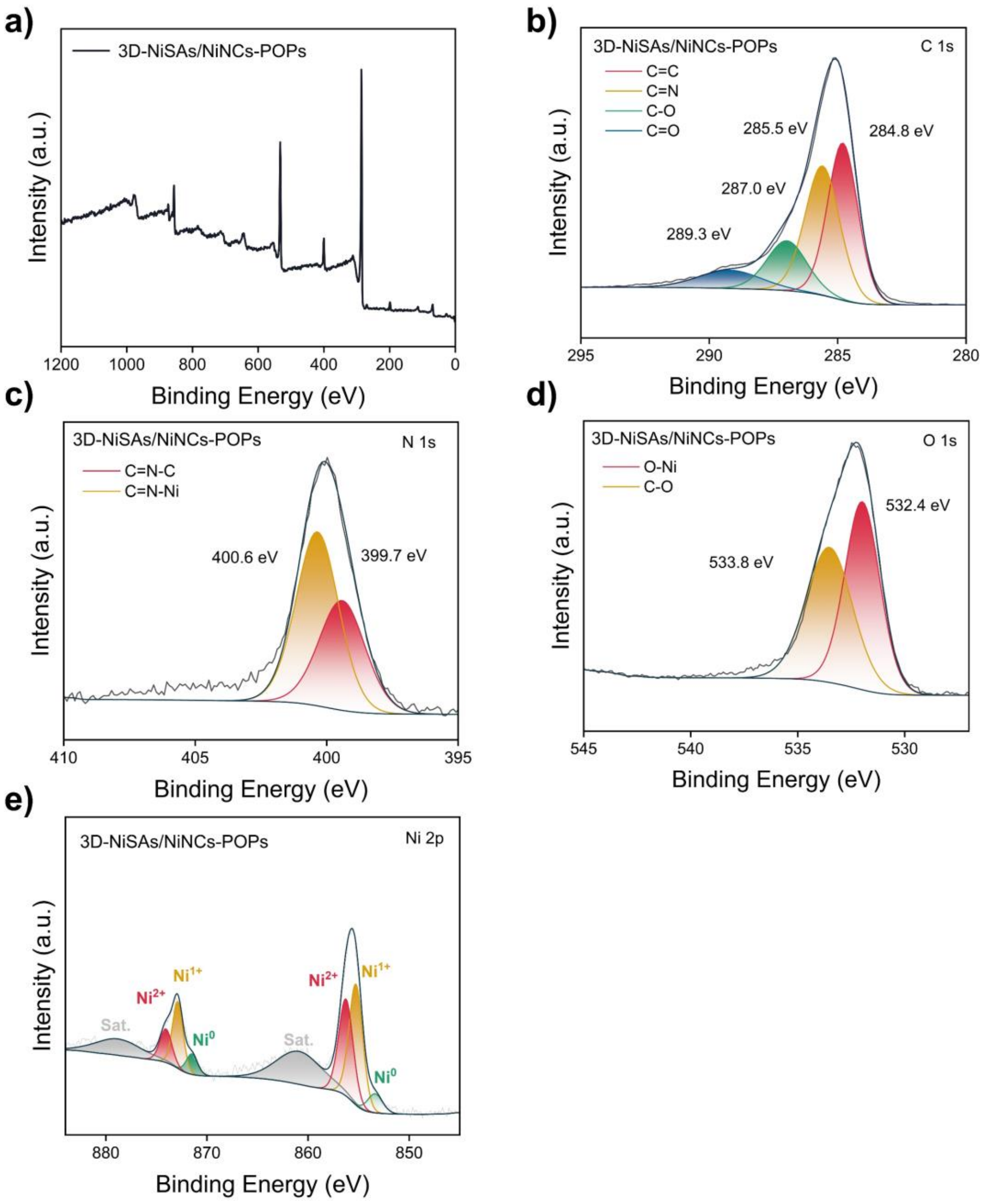

Figure S10. XPS survey spectra of 3D-NiSAs/NiNCs-POPs. a) XPS survey spectrum for 3D-NiSAs/NiNCs-POPs. b, c, d, e). High-resolution XPS spectra of C 1s, N 1s, O 1s and Ni 2p for 3D-NiSAs/NiNCs-POPs.

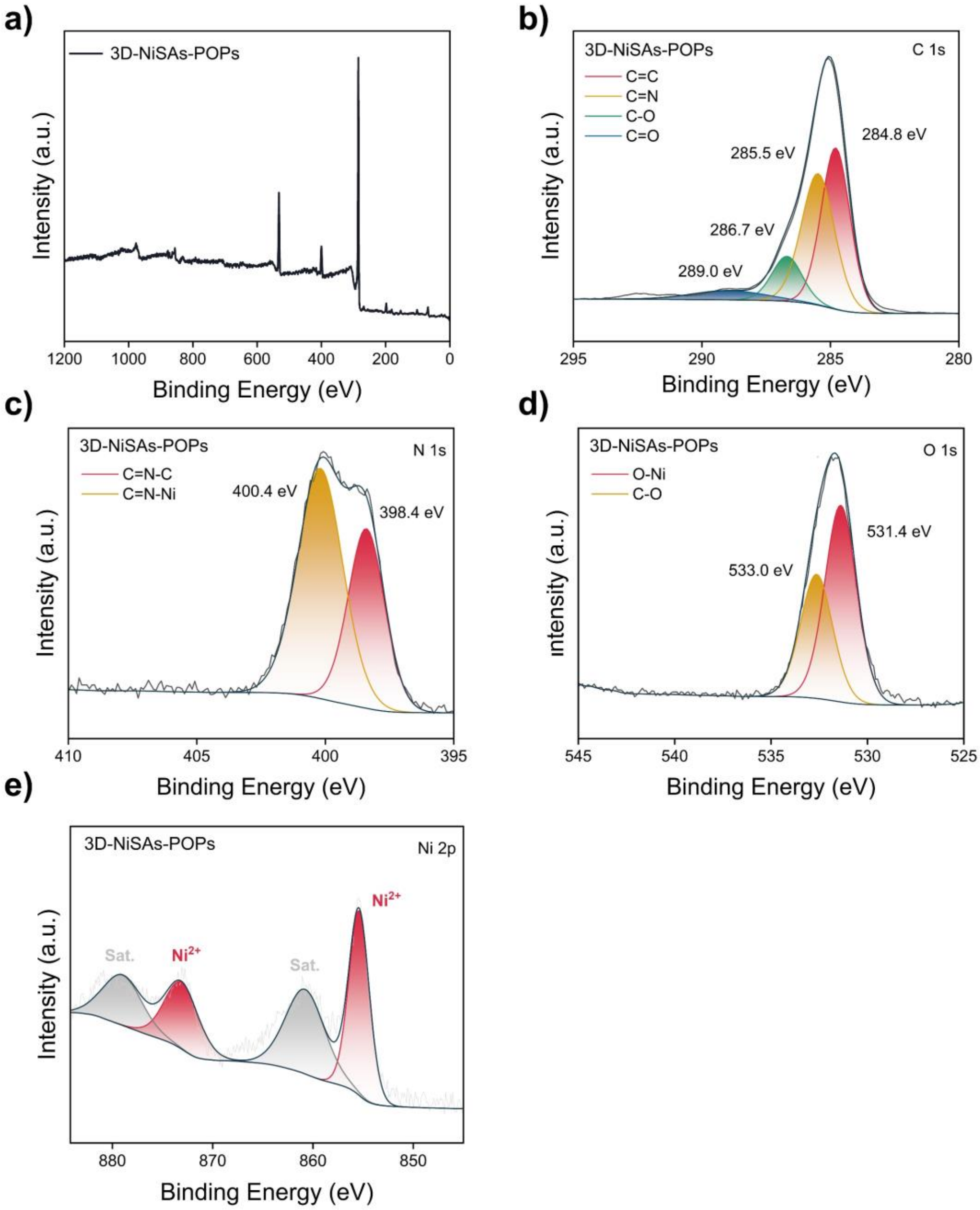

Figure S11. XPS survey spectra of 3D-NiSAs-POPs. a) XPS survey spectrum for 3D-NiSAs-POPs. b, c, d, e) High-resolution XPS spectra of C 1s, N 1s, O 1s and Ni 2p for 3D-NiSACs-POPs.

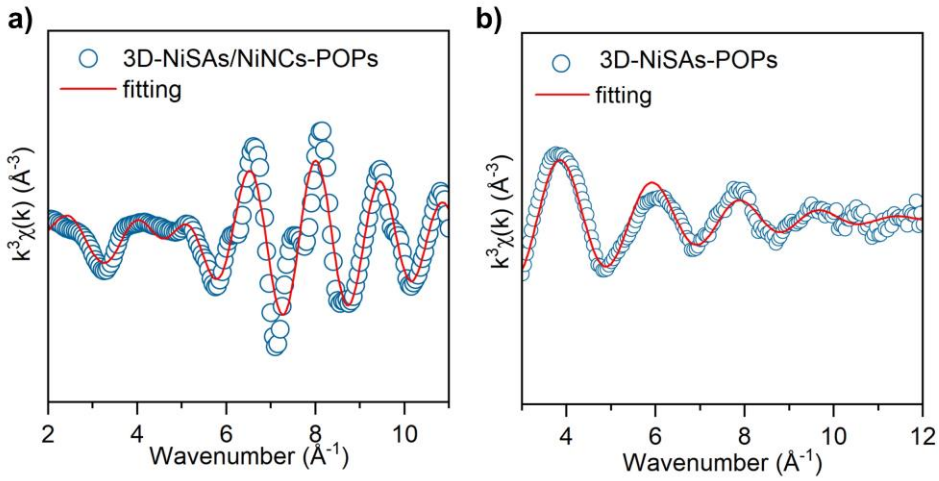

Figure S12. EXFAS fitting curves for a) 3D-NiSAs/NiNCs-POPs and b) 3D-NiSAs-POPs in the k-space.

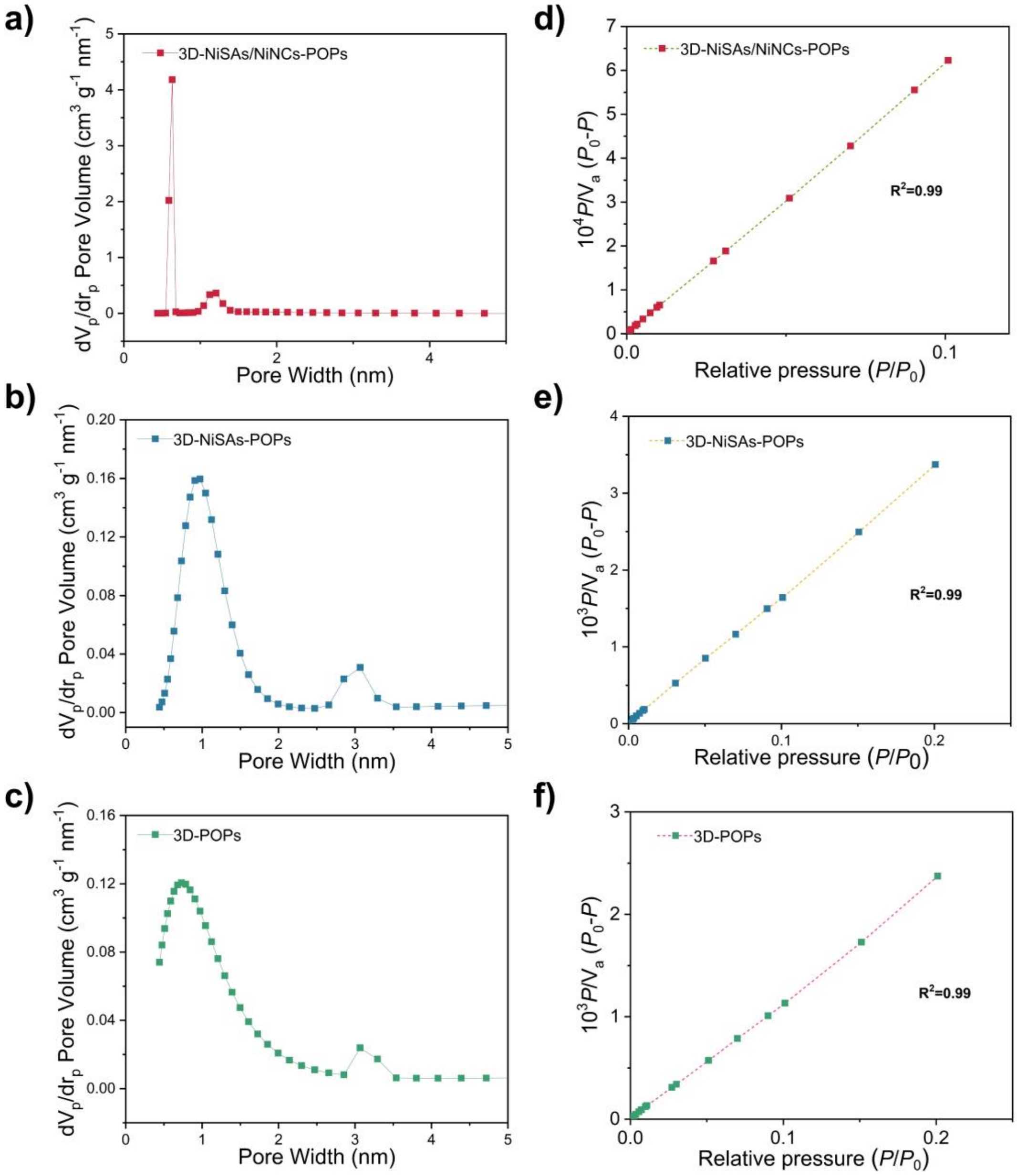

Figure S13. Pore-size distribution profiles of a) 3D-NiSAs/NiNCs-POPs, b) 3D-NiSAs-POPs and c) 3D-POPs. BET plot of d) 3D-NiSAs/NiNCs-POPs, e) 3D-NiSAs-POPs and f) 3D-POPs calculated from N_2 adsorption isotherms at 77 K.

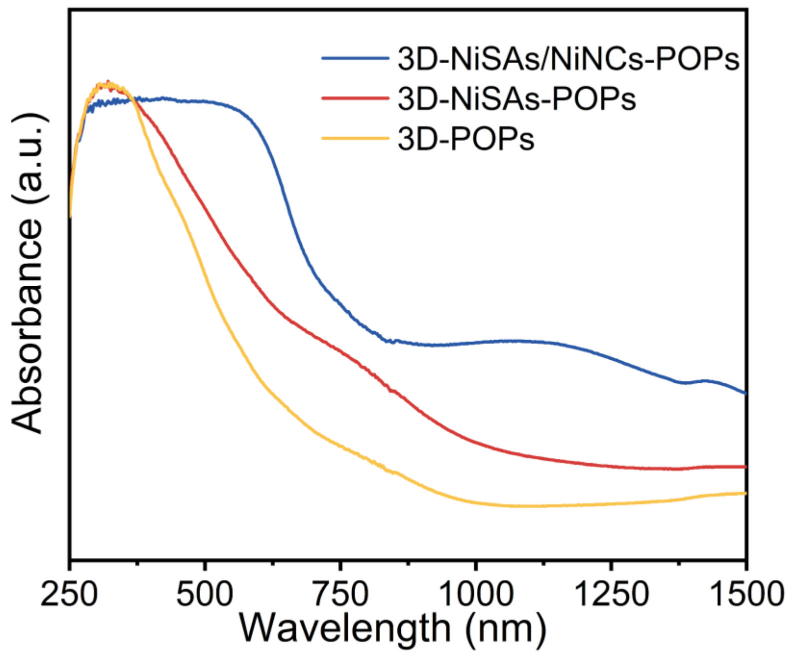

Figure S14. Solid state UV-vis DRS of 3D-NiSAs/NiNCs-POPs (blue), 3D-NiSAs-POPs (red) and 3D-POPs (yellow).

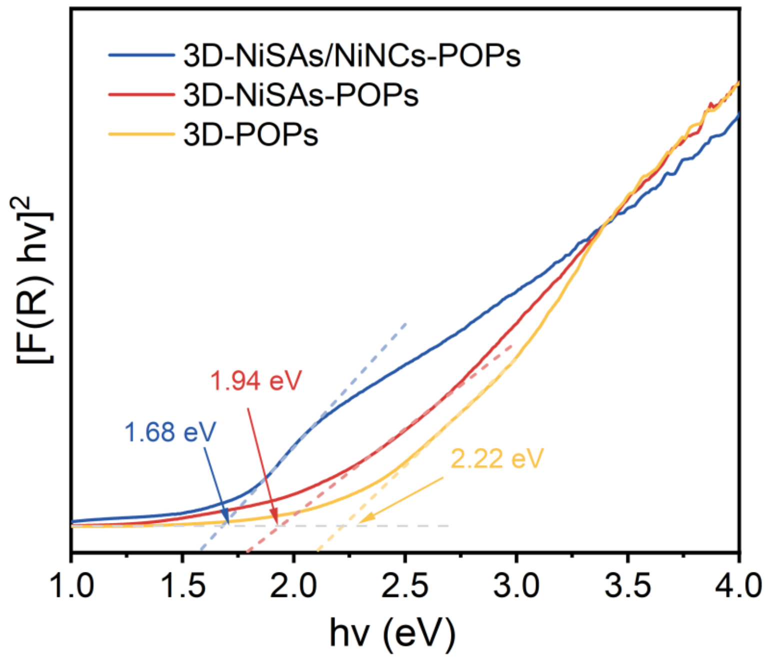

Figure S15. Tauc-plots of 3D-NiSAs/NiNCs-POPs (blue), 3D-NiSAs-POPs (red) and 3D-POPs (yellow) derived from solid state UV-vis diffuse reflectance spectra.

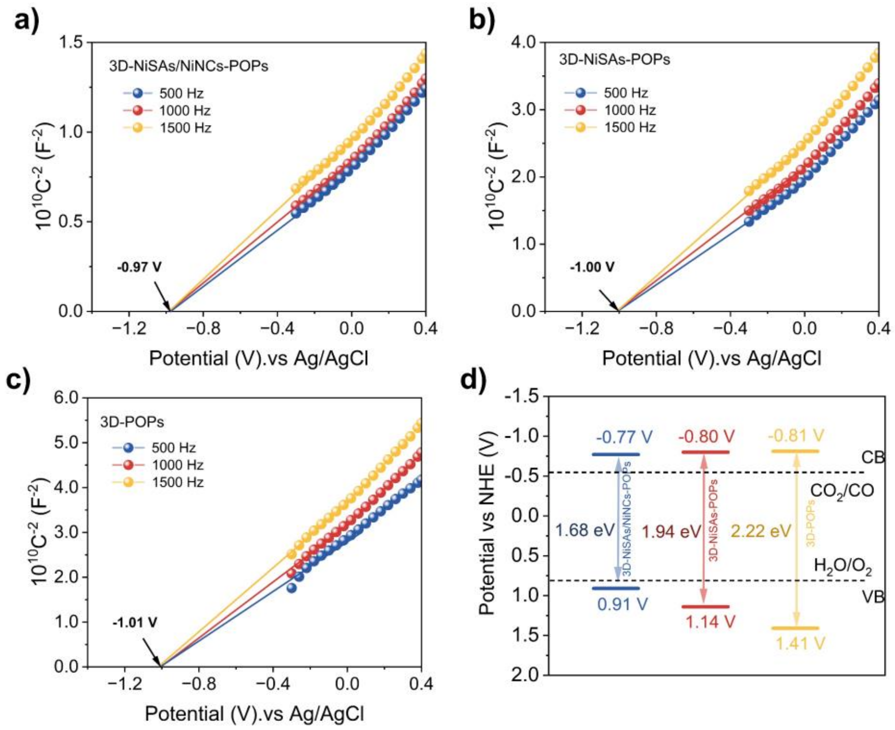

Figure S16. Mott-Schottky plots of a) 3D-NiSAs/NiNCs-POPs, b) 3D-NiSAs-POPs and c) 3D-POPs in 0.5 M Na_2SO_4 aqueous solution. d) Band gap structures of 3D-NiSAs/NiNCs-POPs (blue), 3D-NiSAs-POPs (red) and 3D-POPs (yellow).

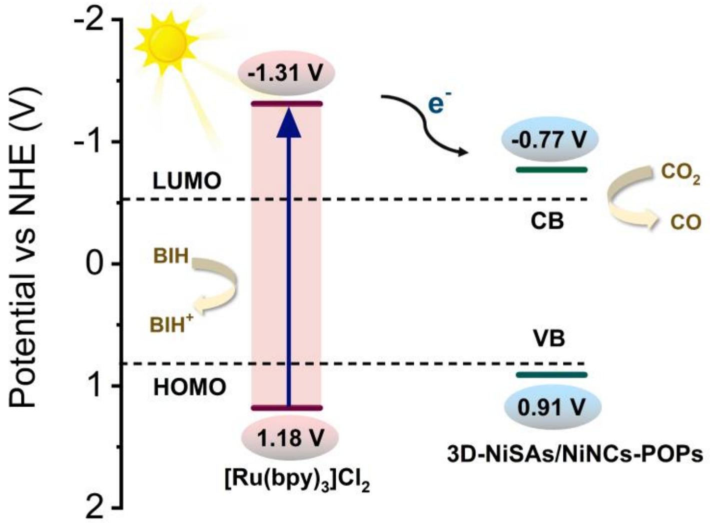

Figure S17. Proposed plausible mechanism of 3D-NiSAs/NiNCs-POPs.

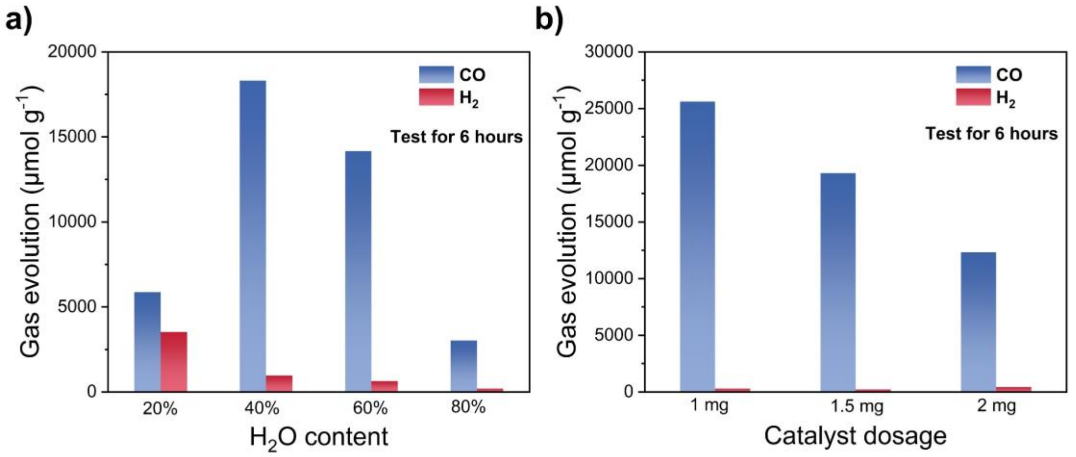

Figure S18. Comparison of the photocatalytic activity of a) The effects of water contents on the evolution of CO_2 and H_2 over 3D-NiSAs/NiNCs-POPs, conditions:1.5mg of 3D-Ni-POPs, 5 mg of $Ru(bpy)_3Cl_2{\cdot}6H_2O$, 25 mg of BIH, the total volume of MeCN and water is 10 mL, LED lamp (400 nm-800 nm), b) The effects of catalyst dosage on the evolution of CO_2 and H_2 over 3D-NiSAs/NiNCs-POP, conditions:5 mg of $Ru(bpy)_3Cl_2{\cdot}6H_2O$, 25 mg of BIH, 6 mL of MeCN, 4 mL of water, LED lamp (400 nm-800 nm).

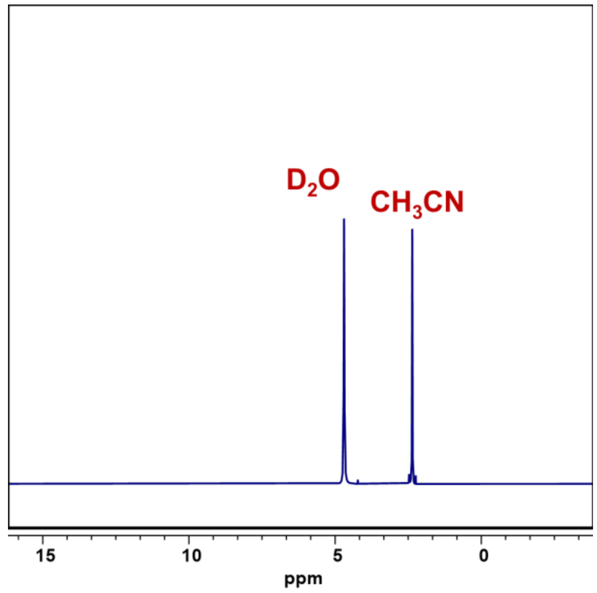

Figure S19. ^{1}H-NMR spectra of the liquid products after a 5 h CO_2 reduction catalyzed by 3D-NiSAs/NiNCs-POPs.

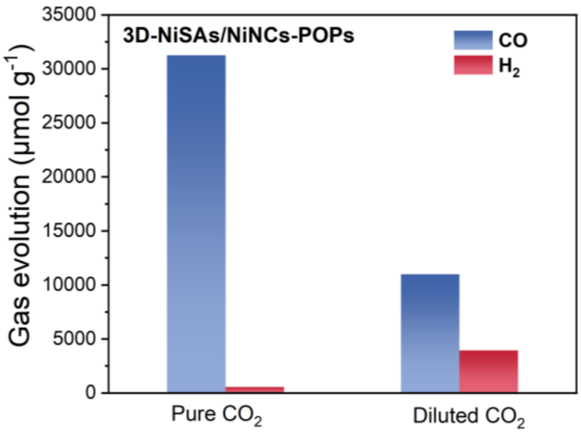

Figure S20. CO_2 photoreduction performance over 3D-NiSAs/NiNCs-POPs in pure CO_2 and diluted CO_2 (15 %).

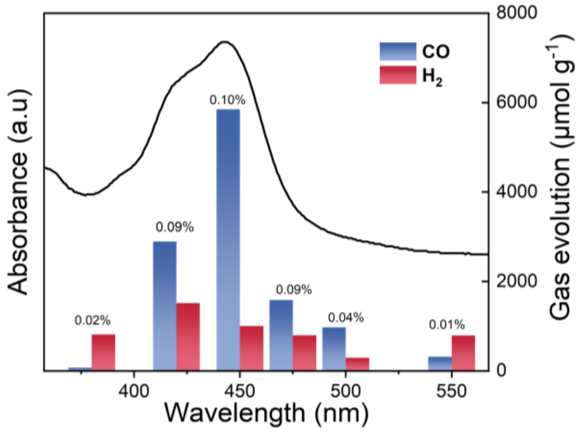

Figure S21. The apparent quantum yields of 3D-NiSAs/NiNCs-POPs and the light absorption spectrum of the Ru photosensitizer.

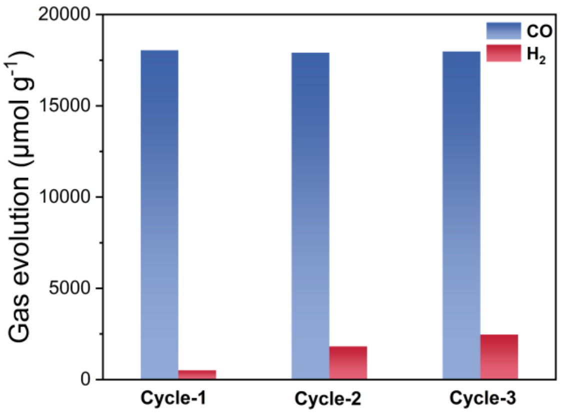

Figure S22. Durability measurements of 3D-NiSAs/NiNCs-POPs (3 h each cycle).

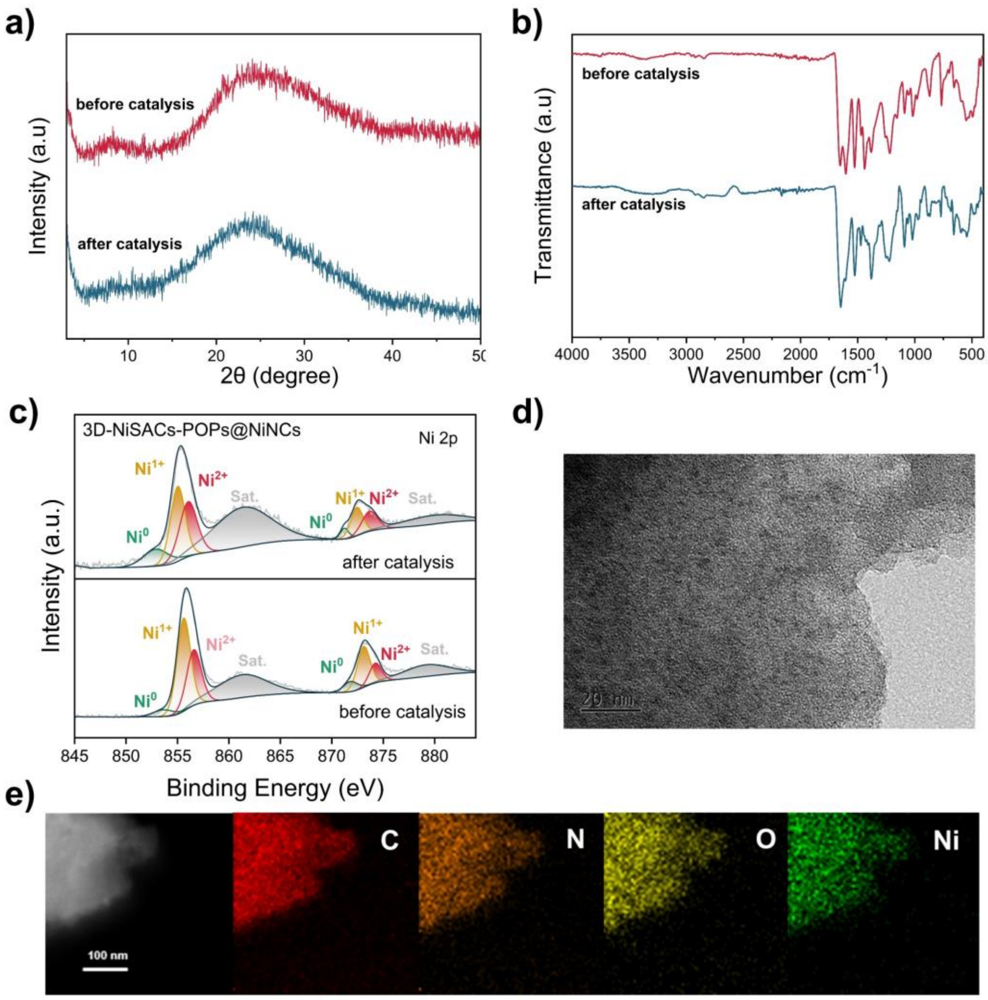

Figure S23. Chemical stability of 3D-NiSAs/NiNCs-POPs before and after 3 cycles of photocatalytic test: a) XRD patterns. b) FT-IR spectra. c) High-resolution XPS spectra of Ni 2p. d, e) TEM images and corresponding elemental mapping of 3D-NiSAs/NiNCs-POPs after 3 cycles of photocatalytic tests.

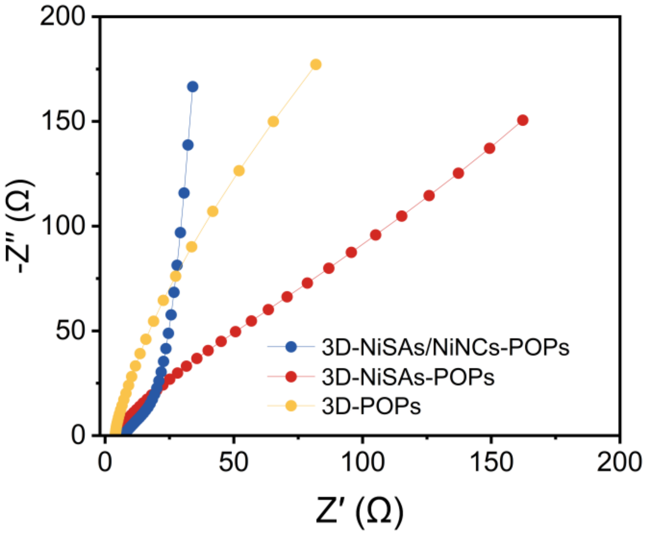

Figure S24. EIS Nyquist plots of 3D-NiSAs/NiNCs-POPs (blue), 3D-NiSAs-POPs (red) and 3D-POPs (yellow).

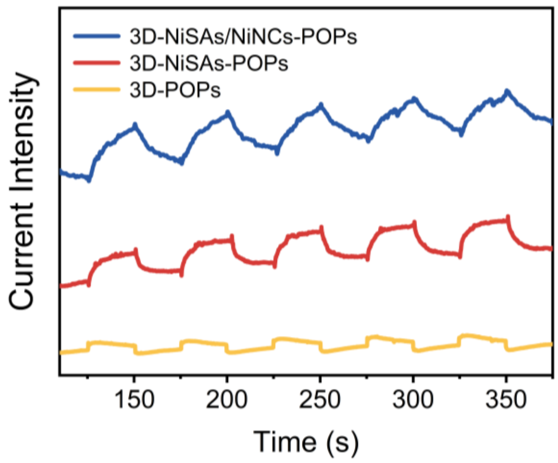

Figure S25. Transient photocurrents under xenon lamp (λ≥ 420 nm) irradiation of 3D-NiSAs/NiNCs-POPs (blue), 3D-NiSAs-POPs (red) and 3D-POPs (yellow).

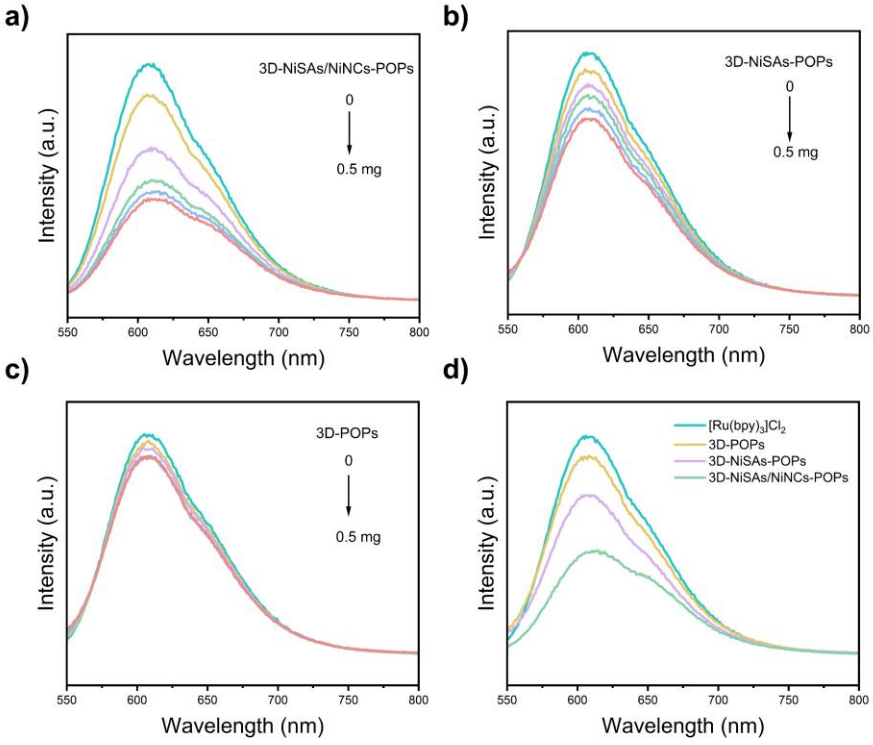

Figure S26. PL spectra of the CO_2 photoreduction system with the addition of increasing amounts of a) 3D-NiSAs/NiNCs-POPs, b) 3D-NiSAs-POPs and c) 3D-POPs. d) PL spectra of the CO_2 photoreduction system without and with the addition of 3D-NiSAs/NiNCs-POPs (green), 3D-NiSAs-POPs (purple) and 3D-POPs(brown) at the same equivalent catalyst loading.

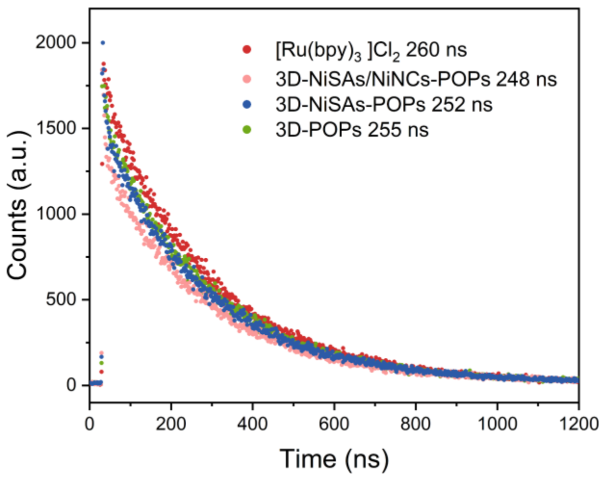

Figure S27. Time-resolved photoluminescence spectra of the CO_2 photoreduction system without (red) and with the addition of 3D-NiSAs/NiNCs-POPs (pink), 3D-NiSAs-POPs (blue) and 3D-POPs (green).

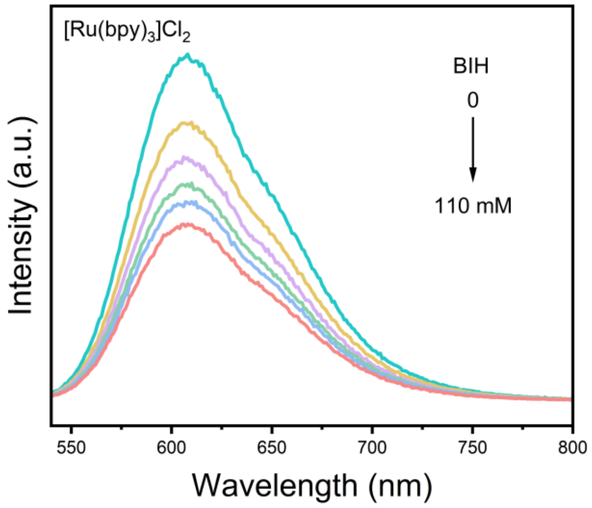

Figure S28. PL spectra of the CO_2 photoreduction system with the addition of increasing amounts of BIH.

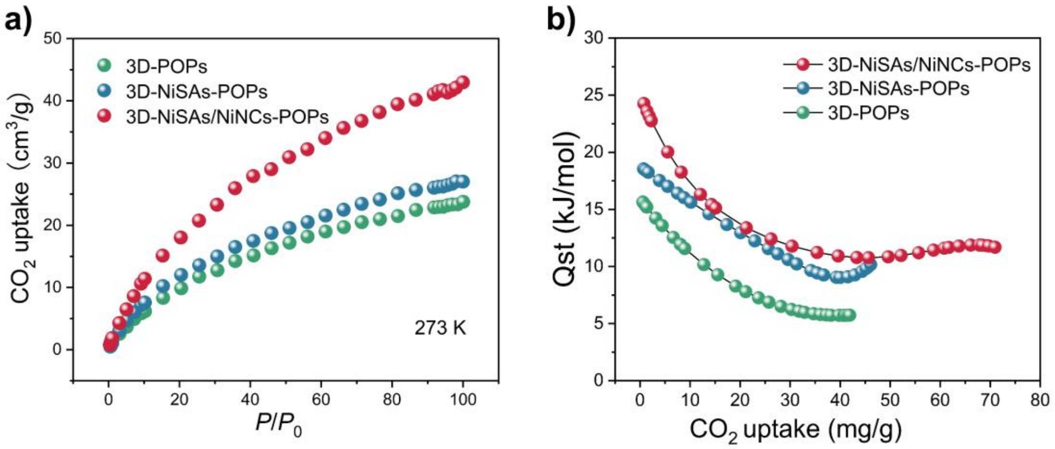

Figure S29. a) CO_2 adsorption isotherms of 3D-NiSAs/NiNCs-POPs, 3D-POPs and 3D-NiSAs-POPs at 273 K. b) The calculated isosteric heat (Qst) of 3D-NiSAs/NiNCs-POPs (24.27 kJ mol^{-1}), 3D-NiSAs-POPs (18.55 kJ mol^{-1}), 3D-POPs (15.65 kJ mol^{-1}).

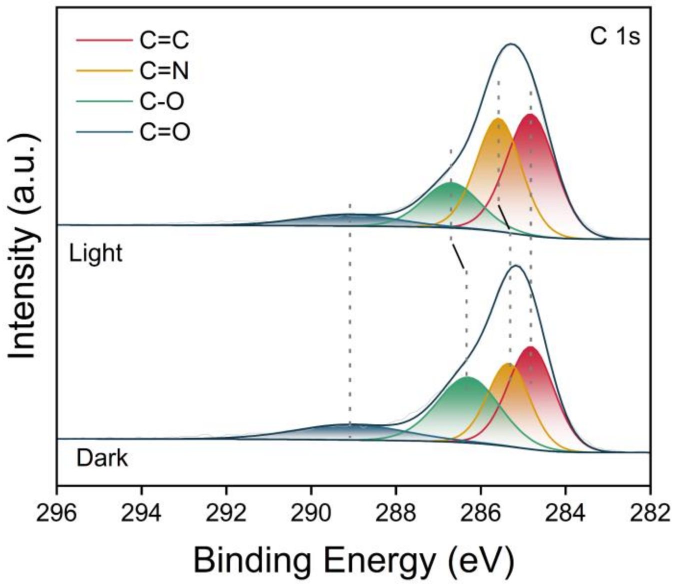

Figure S30. In situ XPS analysis of C 1s in 3D-NiSAs/NiNCs-POPs upon irradiation.

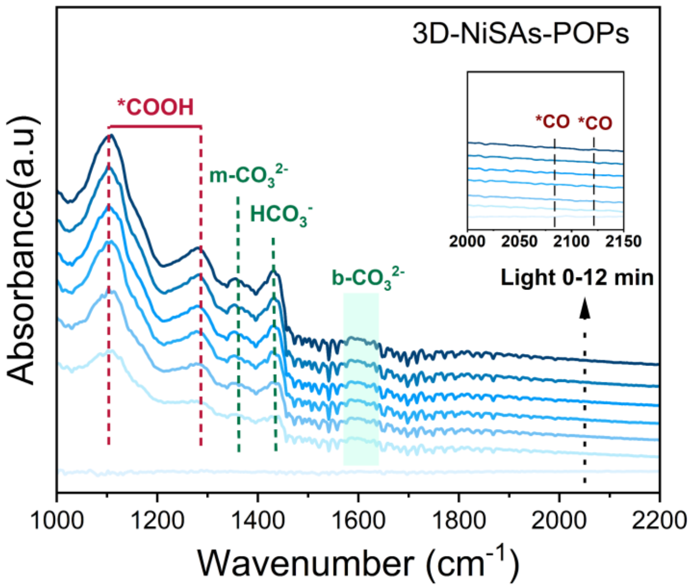

Figure S31. In situ DRIFTS spectra of 3D-NiSAs-POPs.

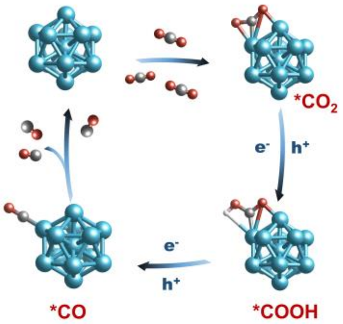

Figure S32. Proposed photocatalytic CO_2 to CO reduction pathways on models of NiNCs.

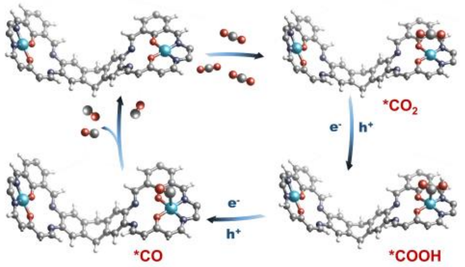

Figure S33. Proposed photocatalytic CO_2 to CO reduction pathways on models of NiSAs.

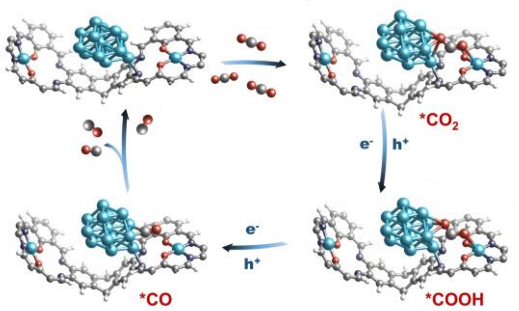

Figure S34. Proposed photocatalytic CO_2 to CO reduction pathways on models of NiSAs/NiNCs-2.

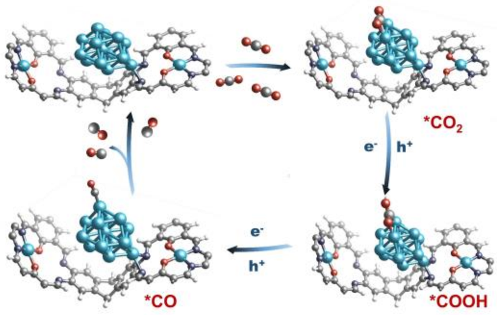

Figure S35. Proposed photocatalytic CO_2 to CO reduction pathways on models of NiSAs/NiNCs-3.

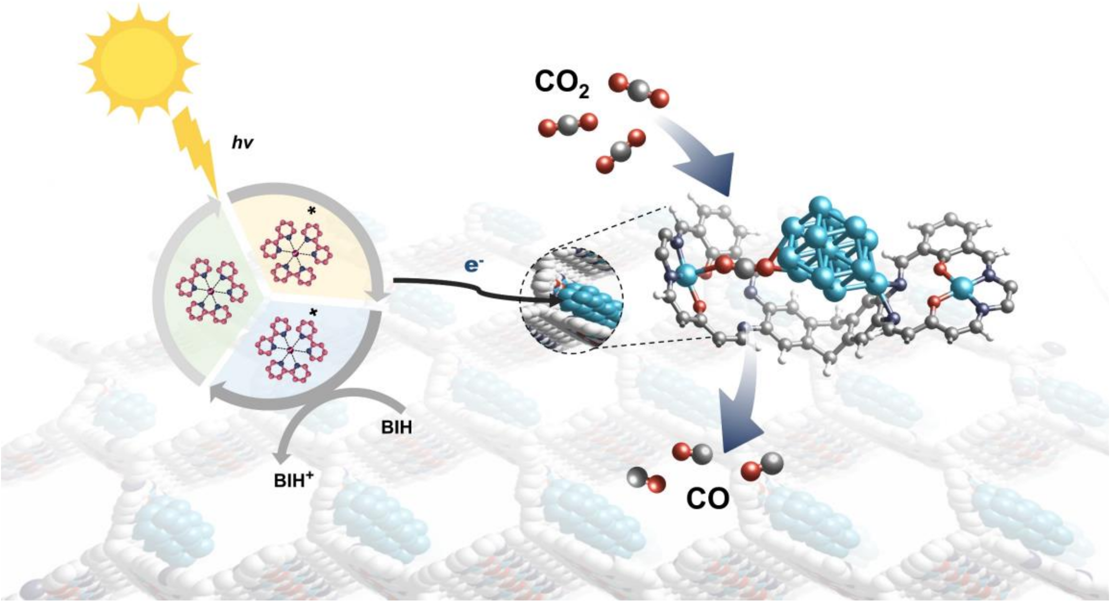

Figure S36. Proposed plausible mechanism of 3D-NiSAs/NiNCs-POPs.

Table S1. The Ni contents in 3D-NiSAs, 3D-NiSAs/NiNCs-POPs and 3D-NiSAs/NiNCs-POPs (post-catalysis).

Sample	Ni content
3D-NiSAs/NiNCs-POPs	10.00 wt%
3D-NiSAs-POPs	3.01 wt%
3D-NiSAs/NiNCs-POPs post-catalysis	8.09 wt%

Table S2. EXAFS fitting parameters at the Ni K-edge for various samples

Sample	Shell	*CN*[a]	*R*(Å)[b]	σ^2(Å^2)[c]	ΔE_0(eV)[d]	*R* factor
Ni foil	Ni-Ni	12*	2.48±0.01	0.0062	6.2	0.0014
NiO	Ni-O	5.8±0.5	2.07±0.01	0.0068	9.4	0.0081
	Ni-Ni	12.5±1.2	2.95±0.01	0.0067	8.7	
NiPc	Ni-N	4.0±0.5	1.87±0.01	0.0026	9.8	0.0152
**3D-NiSAs/NiNCs-POPs**	**Ni-N**	**1.4±0.3**	**1.98±0.01**	**0.0025**	**6.5**	**0.0027**
	**Ni-O**	**1.8±0.3**	**2.10±0.01**	**0.0018**	**9.4**	
	**Ni-Ni**	**4.7±0.1**	**2.48±0.01**	**0.0067**	**7.1**	
**3D-NiSAs -POPs**	**Ni-N**	**2.2±0.3**	**1.99±0.01**	**0.0022**	**-6.5**	**0.0078**
	**Ni-O**	**2.3±0.2**	**2.12±0.01**	**0.0024**	**0.8**	

[a]*CN*, coordination number; [b]*R*, distance between absorber and backscatter atoms; [c]σ^2, Debye-Waller factor to account for both thermal and structural disorders; [d]ΔE_0, inner potential correction; *R* factor indicates the goodness of the fit. S_0^2 was fixed to 0.799, according to the experimental EXAFS fit of Ni foil by fixing CN as the known crystallographic value. A reasonable range of EXAFS fitting parameters: $0.600 < S_0^2 < 1.000$; $CN > 0$; $\sigma^2 > 0$ Å^2; $|\Delta E_0| <$ 15 eV; *R* factor < 0.02.

Table S3. CO_2 geometric parameter of different models.

Model	C-O1	C-O2	Angle
NiSAs	1.17 Å	1.18 Å	179.25°
NiNCs	1.24 Å	1.26 Å	140.03°
NiSAs/NiNCs-1	1.61 Å	1.52 Å	115.06°
NiSAs/NiNCs-2	1.22 Å	1.35 Å	128.06°
NiSAs/NiNCs-3	1.25 Å	1.25 Å	139.93°

Table S4. Comparison of CO_2 photoreduction performance catalyzed by various Ni-based porous photocatalysts.

Photocatalyst	Photosensitizers	Sacrificial agent	Reaction solvent	Light Condition	CO (μmol g^{-1} h^{-1})	CO selectivity	Ref.
3D-NiSAs/NiNCs-POPs	$Ru(bpy)_3Cl_2 \cdot 6H_2O$	BIH	$MeCN/H_2O$	300 W Xe lamp (Cut 420 nm)	6240	98%	**This work**
Ni-COF	$Ru(bpy)_3]I_2 \cdot 6H_2O$	TEOA	$MeCN/H_2O$	White LED (400 nm- 800 nm)	5310	95%	[6]
Ni-TP-CON	$Ru(bpy)_3Cl_2 \cdot 6H_2O$	TEOA	$MeCN/H_2O$	300 W Xe lamp (Cut 420 nm)	4360	95%	[7]
Ni/SOM-ZIF-8	$Ru(bpy)_3Cl_2 \cdot 6H_2O$	TEOA	$MeCN/H_2O$	300 W Xe lamp (Cut 400 nm)	4200	94%	[8]
TTPP-Ni-n	$Ru(bpy)_3Cl_2 \cdot 6H_2O$	TIPA	$MeCN/H_2O$	300 W Xe lamp (Cut 420 nm)	3900	98%	[9]
Ni-MOL-100	$Ru(bpy)_3Cl_2 \cdot 6H_2O$	TEOA	$MeCN/H_2O$	300 W Xe lamp (Cut 420 nm)	2972	97%	[10]
H-COF-Ni	$Ru(bpy)_3Cl_2 \cdot 6H_2O$	TEOA	$MeCN/H_2O$	300 W Xe lamp (Cut 420 nm)	2847	96%	[11]
Bpy-PDI-Ni	$Ru(bpy)_3Cl_2 \cdot 6H_2O$	TEOA	$MeCN/H_2O$	White LED (400 nm- 800 nm)	2262	100%	[12]
NiPc-NiPOP	$Ru(bpy)_3Cl_2 \cdot 6H_2O$	TEOA	$MeCN/H_2O$	White LED (400 nm- 800 nm)	1940	96%	[13]
NiPor-BDOB	$Ru(bpy)_3Cl_2 \cdot 6H_2O$	TEA	$MeCN/H_2O$	300 W Xe lamp	1770	97%	[14]
Ni@TPHH-COF	$Ru(bpy)_3Cl_2 \cdot 6H_2O$	TEOA	$MeCN/H_2O$	300 W Xe lamp (Cut 420 nm)	1610	96%	[15]
Ni-TpBpy	$Ru(bpy)_3Cl_2 \cdot 6H_2O$	TEOA	$MeCN/H_2O$	300 W Xe lamp (Cut 420 nm)	811	97%	[16]
Ni-MOF(H_2O)	$Ru(bpy)_3Cl_2 \cdot 6H_2O$	TEOA	$MeCN/H_2O$	White LED (400 nm- 800 nm)	666	95%	[17]
NiP-TPE-COF	$Ru(bpy)_3Cl_2 \cdot 6H_2O$	TEOA	$MeCN/H_2O$	300 W Xe lamp (Cut 420 nm)	525	93%	[18]
Ni-PCD@TD-COF	$Ru(bpy)_3Cl_2 \cdot 6H_2O$	TEOA	$MeCN/H_2O$	300 W Xe lamp (Cut 420 nm)	478	98%	[19]
Ni_xCo_{1-x}-MOFs	$Ru(bpy)_3Cl_2 \cdot 6H_2O$	TEOA	DMF/H_2O	300 W Xe lamp (Cut 400 nm)	277	85%	[20]

References

[1] J. P. Perdew, K. Burke, M. Ernzerhof, *Phys Rev Lett.* **1996**, *77*, 3865.

[2] B. Hammer, L. B. Hansen, J. K. Nørskov, *Physical Review B.* **1999**, *59*, 7413.

[3] P. E. Blöchl, *Physical Review B.* **1994**, *50*, 17953.

[4] G. Kresse, D. Joubert, *Physical Review B.* **1999**, *59*, 1758.

[5] H. J. Monkhorst, J. D. Pack, *Physical Review B.* **1976**, *13*, 5188.

[6] B. Han, X. Ou, Z. Zhong, S. Liang, H. Deng, Z. Lin, *Small.* **2020**, *16*, 2002985.

[7] H. Lv, P. Li, X. Li, A. Chen, R. Sa, H. Zhu, R. Wang, *Chem. Eng. J.* **2023**, *451*, 138745.

[8] Z. Liu, Z. Chen, M. Li, J. Li, W. Zhuang, X. Yang, S. Wu, J. Zhang, *ACS Catal.* **2023**, *13*, 6630.

[9] X. Ding, B. Yu, B. Han, H. Wang, T. Zheng, B. Chen, J. Wang, Z. Yu, T. Sun, X. Fu, D. Qi, J. Jiang, *ACS Appl. Mater. Interfaces.* **2022**, *14*, 8048.

[10] W. Yang, H. J. Wang, R. R. Liu, J. W. Wang, C. Zhang, C. Li, D. C. Zhong, T. B. Lu, *Angew. Chem. Int. Ed.* **2020**, *60*, 409.

[11] S. Yang, R. Sa, H. Zhong, H. Lv, D. Yuan, R. Wang, *Adv. Funct. Mater.* **2022**, *32*, 2110694.

[12] S. Liang, X. Zhong, Z. Zhong, H. Deng, W.-Y. Wong, *Appl. Catal. B Environ.* **2023**, *337*, 122958.

[13] X. Y. Dong, Y. N. Si, Q. Y. Wang, S. Wang, S. Q. Zang, *Adv. Mater.* **2021**, *33*, 2101568.

[14] S. Suleman, K. Sun, Y. Zhao, X. Guan, Z. Lin, Z. Meng, H.-L. Jiang, *CCS Chem.* **2023**, *0*, 1.

[15] M. Dong, J. Zhou, J. Zhong, H. T. Li, C. Y. Sun, Y. D. Han, J. N. Kou, Z. H. Kang, X. L. Wang, Z. M. Su, *Adv. Funct. Mater.* **2021**, *32*, 2110136.

[16] W. Zhong, R. Sa, L. Li, Y. He, L. Li, J. Bi, Z. Zhuang, Y. Yu, Z. Zou, *J. Am. Chem. Soc.* **2019**, *141*, 7615.

[17] K. Song, S. Liang, X. Zhong, M. Wang, X. Mo, X. Lei, Z. Lin, *Appl. Catal. B Environ* **2022**, *309*, 121232.

[18] H. Lv, R. Sa, P. Li, D. Yuan, X. Wang, R. Wang, *Science China Chemistry.* **2020**, *63*, 1289.

[19] H. Zhong, R. Sa, H. Lv, S. Yang, D. Yuan, X. Wang, R. Wang, *Adv. Funct. Mater.* **2020**,

30, 2002654.

[20] H. Yang, D. Zhang, Y. Luo, W. Yang, X. Zhan, W. Yang, H. Hou, *Small* **2022**, *18*, 2202939.
